# Supplementary material for: The Sailboat Activity: An Interactive, Visually Engaging Approach to Design and Assess Health Profession Education Research Projects
Source: MedEdPORTAL. 2025 May 2;21:11520. doi: 10.15766/mep_2374-8265.11520 (PMC12046060; doi:10.15766/mep_2374-8265.11520)
Supplement: Supplementary file 1 — Sailboat Template.pptxPreworkshop Assignment Instructions.docxPreworkshop Survey.docxFacilitator Guide.docxSailboat Activity Session Slides.pptxCollaborative Working Area.pptxPostworkshop Survey.docxAction Plan Scoring Rubric.docx [file mep_2374-8265.11520-s001.zip › G. Postworkshop Survey.docx]

**Post-Workshop Survey**

Introduction

Dear All,

We are curious to learn about your experience with the *Sailboat Activity*. Your responses will help us to make changes in the design of this session for future iterations.

This survey should take you less than *three minutes* to complete, your participation is voluntarily and anonymous.

Thank you for providing us feedback!

Questions

1. How useful was the Sailboat Activity to outline your research idea?

| 〇 | 〇 | 〇 | 〇 | 〇 |
| --- | --- | --- | --- | --- |
| Not at all useful | Slightly useful | Moderately useful | Very useful | Extremely useful |

1. How useful was the Sailboat Activity to help you articulate the barriers for your research project?

| 〇 | 〇 | 〇 | 〇 | 〇 |
| --- | --- | --- | --- | --- |
| Not at all useful | Slightly useful | Moderately useful | Very useful | Extremely useful |

1. How useful was the Sailboat Activity to help you articulate the strengths for your research project?

| 〇 | 〇 | 〇 | 〇 | 〇 |
| --- | --- | --- | --- | --- |
| Not at all useful | Slightly useful | Moderately useful | Very useful | Extremely useful |

1. How useful was the Sailboat Activity to help you articulate the weaknesses for your research project?

| 〇 | 〇 | 〇 | 〇 | 〇 |
| --- | --- | --- | --- | --- |
| Not at all useful | Slightly useful | Moderately useful | Very useful | Extremely useful |

1. How useful was the Sailboat Activity to help you appraise your research project?

| 〇 | 〇 | 〇 | 〇 | 〇 |
| --- | --- | --- | --- | --- |
| Not at all useful | Slightly useful | Moderately useful | Very useful | Extremely useful |

1. How useful was the Sailboat Activity to help you appraise your partner’s research project?

| 〇 | 〇 | 〇 | 〇 | 〇 |
| --- | --- | --- | --- | --- |
| Not at all useful | Slightly useful | Moderately useful | Very useful | Extremely useful |

1. How useful was the Sailboat Activity to identify ways to improve your research project?

| 〇 | 〇 | 〇 | 〇 | 〇 |
| --- | --- | --- | --- | --- |
| Not at all useful | Slightly useful | Moderately useful | Very useful | Extremely useful |

1. How useful was the Sailboat Activity to help you analyze the alignment of the main elements (problem, research question, objectives, type of study, variables, population and sample, and outcome measures) of your research project?

| 〇 | 〇 | 〇 | 〇 | 〇 |
| --- | --- | --- | --- | --- |
| Not at all useful | Slightly useful | Moderately useful | Very useful | Extremely useful |

1. How useful was the Sailboat Activity to help you recognize the iterative nature of designing a research project?

| 〇 | 〇 | 〇 | 〇 | 〇 |
| --- | --- | --- | --- | --- |
| Not at all useful | Slightly useful | Moderately useful | Very useful | Extremely useful |

1. How useful was the Sailboat Activity to help you articulate actions steps in SMART format?

| 〇 | 〇 | 〇 | 〇 | 〇 |
| --- | --- | --- | --- | --- |
| Not at all useful | Slightly useful | Moderately useful | Very useful | Extremely useful |

1. Overall, how helpful was the Sailboat Activity in your learning?

| 〇 | 〇 | 〇 | 〇 | 〇 |
| --- | --- | --- | --- | --- |
| Not at all useful | Slightly useful | Moderately useful | Very useful | Extremely useful |

1. As you look at your Sailboat ***now*** *(after participating in today's Sailboat activity)*, how confident do you feel about the barriers of your research project?

| 〇 | 〇 | 〇 | 〇 | 〇 |
| --- | --- | --- | --- | --- |
| Not at all confident | Slightly confident | Moderately confident | Quite confident | Extremely confident |

1. As you look at your Sailboat ***now*** *(after participating in today's Sailboat activity),* how confident do you feel about the goal(s) of your research project?

| 〇 | 〇 | 〇 | 〇 | 〇 |
| --- | --- | --- | --- | --- |
| Not at all confident | Slightly confident | Moderately confident | Quite confident | Extremely confident |

1. As you look at your Sailboat ***now*** *(after participating in today's Sailboat activity),* how confident do you feel about the strengths of your research project?

| 〇 | 〇 | 〇 | 〇 | 〇 |
| --- | --- | --- | --- | --- |
| Not at all confident | Slightly confident | Moderately confident | Quite confident | Extremely confident |

1. As you look at your Sailboat ***now*** *(after participating in today's Sailboat activity),* how confident do you feel about the weaknesses of your research project?

| 〇 | 〇 | 〇 | 〇 | 〇 |
| --- | --- | --- | --- | --- |
| Not at all confident | Slightly confident | Moderately confident | Quite confident | Extremely confident |

1. As you look at your Sailboat ***now*** *(after participating in today's Sailboat activity),* how confident do you feel about your project’s main elements (problem, research question, objectives, type of study, variables, population and sample, and outcome measures)?

| 〇 | 〇 | 〇 | 〇 | 〇 |
| --- | --- | --- | --- | --- |
| Not at all confident | Slightly confident | Moderately confident | Quite confident | Extremely confident |

1. As you look at your Sailboat ***now*** *(after participating in today's Sailboat activity)*, how confident do you feel about the alignment of the main elements (problem, research question, objectives, type of study, variables, population and sample, and outcome measures) of your research project?

| 〇 | 〇 | 〇 | 〇 | 〇 |
| --- | --- | --- | --- | --- |
| Not at all confident | Slightly confident | Moderately confident | Quite confident | Extremely confident |

1. As you look at your Sailboat ***now*** *(after participating in today's Sailboat activity),* how confident do you feel about the next steps in your project development?

| 〇 | 〇 | 〇 | 〇 | 〇 |
| --- | --- | --- | --- | --- |
| Not at all confident | Slightly confident | Moderately confident | Quite confident | Extremely confident |

1. What were the strengths of the *Sailboat activity*?
2. What could have gone better in the *Sailboat activity*?
