# Supplementary material for: The Sailboat Activity: An Interactive, Visually Engaging Approach to Design and Assess Health Profession Education Research Projects
Source: MedEdPORTAL. 2025 May 2;21:11520. doi: 10.15766/mep_2374-8265.11520 (PMC12046060; doi:10.15766/mep_2374-8265.11520)
Supplement: Supplementary file 1 — Sailboat Template.pptxPreworkshop Assignment Instructions.docxPreworkshop Survey.docxFacilitator Guide.docxSailboat Activity Session Slides.pptxCollaborative Working Area.pptxPostworkshop Survey.docxAction Plan Scoring Rubric.docx [file mep_2374-8265.11520-s001.zip › H. Action Plan Scoring Rubric.docx]

**Action Plan Scoring Rubric**

The Action Plan Scoring Rubric assists faculty in evaluating the action steps crafted by participants during the Sailboat activity.

**Scoring Criteria**

1. Quality of Objective: Inclusion of SMART criteria

1 point = Vague action step without clear connection to the SMART criteria

2 points = Some of the SMART elements are represented but not all of them

3 points = All SMART elements are included

1. Alignment with the project’s weaknesses (anchors or iceberg)

1 point = no alignment between the action plan and the project’s weakness(es)

2 points = action plan is aligned with the project weaknesses

| Participant’s action plan | Quality | Alignment |
| --- | --- | --- |
|  |  |  |
|  |  |  |
|  |  |  |

SMART criteria:

*S = Specific – Is the action plan focused on a clear aim?*

*M = Measurable: Could someone determine whether you have completed your action plan?*

*A = Achievable: Do you have the resources and capabilities to complete your action plan?*

*R = Relevant: Does the action plan align with the challenge/weakness you identified?*

*T = Time-bound: Does the action plan have a deadline?*
