# Supplementary material for: The Sailboat Activity: An Interactive, Visually Engaging Approach to Design and Assess Health Profession Education Research Projects
Source: MedEdPORTAL. 2025 May 2;21:11520. doi: 10.15766/mep_2374-8265.11520 (PMC12046060; doi:10.15766/mep_2374-8265.11520)
Supplement: Supplementary file 1 — Sailboat Template.pptxPreworkshop Assignment Instructions.docxPreworkshop Survey.docxFacilitator Guide.docxSailboat Activity Session Slides.pptxCollaborative Working Area.pptxPostworkshop Survey.docxAction Plan Scoring Rubric.docx [file mep_2374-8265.11520-s001.zip › B. Preworkshop Assignment Instructions.docx]

**Pre-Workshop Instructions: Sailboat activity**

Please outline a concept of a Health Profession Education Research (HPER) plan by following the steps described below. We invite you to draw or annotate a ‘Sailboat’ as a visual representation of your HPER plan idea. You can draw your sailboat on paper or electronically using any computer or tablet program you wish (e.g. PowerPoint).

1. Draw a sailboat on paper, whiteboard, or electronically. You can also choose to use one of the templates we included.
2. The sailboat is your project, please name it as you wish.
3. Describe what characterizes your sailboat (your project): add details on the body of the boat (Research Question), sails (Research Problem, Conditions), windows (Variables, Population and Sample, Outcome measures), flag (Research Design). Please note your sample selection process. Consider using the PICOTS (Population, Intervention, Control, Outcomes, Time, Setting), I-SMART (Important, Specific, Measurable, Achievable, Relevant, Timely) or FINER (Feasible, Important and Interesting, Ethical, Relevant) criteria to evaluate the completeness of your research question(s). Use the Kirkpatrick evaluation model^1^ when you identify the outcome measures (Level 1: reaction, Level 2: learning, Level 3: behavior change, Level 4: patient impact).
4. Draw an island that represents the final goal (significance) of your project. This is the place where you can answer the “So what?” question.
5. Draw water around the island and the sailboat. The water represents the research site, please describe it briefly.
6. Draw an iceberg, which represents the major problem that might arise during your project. This is something that can ruin your project (e.g., low sample size, lack of institutional support).
7. Draw an anchor from the sailboat that goes into the water. The anchor represents the obstacles slowing the movement of your sailboat. Think about barriers that could hold you back in the development of your project (e.g., lack of access to learners, faculty, technology). The lower they are under water, the stronger they are.
8. Finally, draw some wind that represents positive elements that push your sailboat to the island. This is also a place where you can briefly describe the theoretical or conceptual framework of the project.

Please review your partner’s Sailboat before the Sailboat activity workshop. Consider the following questions when you think about your partner’s Sailboat. Make notes of the points you wish to discuss with your partner during our Sailboat activity.

- How can you characterize the HPER project? What is/are the research question(s) and outcome measures?
- Is there a theoretical or conceptual framework to situate the HPER project?
- Does the research design align with the research question(s)?
- What are the strengths and weaknesses of the project plan?
- Does the project seem feasible? What part of the project may need additional support?

^1^ Kirkpatrick JD, Kirkpatrick WK. Chapter 2: New World Kirkpatrick Model. In: Kirkpatrick JD, Kirkpatrick WK. *Kirkpatrick's four levels of training evaluation.* *(First edition).* Association for Talent Development; 2016.
