# Supplementary material for: The Sailboat Activity: An Interactive, Visually Engaging Approach to Design and Assess Health Profession Education Research Projects
Source: MedEdPORTAL. 2025 May 2;21:11520. doi: 10.15766/mep_2374-8265.11520 (PMC12046060; doi:10.15766/mep_2374-8265.11520)
Supplement: Supplementary file 1 — Sailboat Template.pptxPreworkshop Assignment Instructions.docxPreworkshop Survey.docxFacilitator Guide.docxSailboat Activity Session Slides.pptxCollaborative Working Area.pptxPostworkshop Survey.docxAction Plan Scoring Rubric.docx [file mep_2374-8265.11520-s001.zip › C. Preworkshop Survey.docx]

**Pre-Workshop Survey**

Introduction

Dear All,

We are curious to learn about your experience with the Sailboat Activity. Your responses will help us to make changes in the design of this session for future iterations.

This survey should take you less than 2 minutes to complete, your participation is voluntarily and anonymous.

Thank you for providing us feedback!

Questions

1. As you look at your Sailboat ***now***, how confident do you feel about the barriers of your research project?

| 〇 | 〇 | 〇 | 〇 | 〇 |
| --- | --- | --- | --- | --- |
| Not at all confident | Slightly confident | Moderately confident | Quite confident | Extremely confident |

1. As you look at your Sailboat ***now***, how confident do you feel about the goal(s) of your research project?

| 〇 | 〇 | 〇 | 〇 | 〇 |
| --- | --- | --- | --- | --- |
| Not at all confident | Slightly confident | Moderately confident | Quite confident | Extremely confident |

1. As you look at your Sailboat ***now***, how confident do you feel about the strengths of your research project?

| 〇 | 〇 | 〇 | 〇 | 〇 |
| --- | --- | --- | --- | --- |
| Not at all confident | Slightly confident | Moderately confident | Quite confident | Extremely confident |

1. As you look at your Sailboat ***now***, how confident do you feel about the weaknesses of your research project?

| 〇 | 〇 | 〇 | 〇 | 〇 |
| --- | --- | --- | --- | --- |
| Not at all confident | Slightly confident | Moderately confident | Quite confident | Extremely confident |

1. As you look at your Sailboat ***now***, how confident do you feel about your project’s main elements (problem, research question, objectives, type of study, variables, population and sample, and outcome measures)?

| 〇 | 〇 | 〇 | 〇 | 〇 |
| --- | --- | --- | --- | --- |
| Not at all confident | Slightly confident | Moderately confident | Quite confident | Extremely confident |

1. As you look at your Sailboat ***now***, how confident do you feel about the alignment of the main elements (problem, research question, objectives, type of study, variables, population and sample, and outcome measures) of your research project?

| 〇 | 〇 | 〇 | 〇 | 〇 |
| --- | --- | --- | --- | --- |
| Not at all confident | Slightly confident | Moderately confident | Quite confident | Extremely confident |

1. As you look at your Sailboat ***now***, how confident do you feel about the next steps in your project development?

| 〇 | 〇 | 〇 | 〇 | 〇 |
| --- | --- | --- | --- | --- |
| Not at all confident | Slightly confident | Moderately confident | Quite confident | Extremely confident |
