# Supplementary material for: The Sailboat Activity: An Interactive, Visually Engaging Approach to Design and Assess Health Profession Education Research Projects
Source: MedEdPORTAL. 2025 May 2;21:11520. doi: 10.15766/mep_2374-8265.11520 (PMC12046060; doi:10.15766/mep_2374-8265.11520)
Supplement: Supplementary file 1 — Sailboat Template.pptxPreworkshop Assignment Instructions.docxPreworkshop Survey.docxFacilitator Guide.docxSailboat Activity Session Slides.pptxCollaborative Working Area.pptxPostworkshop Survey.docxAction Plan Scoring Rubric.docx [file mep_2374-8265.11520-s001.zip › D. Facilitator Guide.docx]

**Sailboat Activity Facilitator Guide**

The present guide includes the educational objectives and lists the questions and discussion points for the detailed session plan. Each discussion point is followed by parentheses including the relevant educational objective(s). We created the discussion points bason on our experience with the Sailboat activity session. The suggested discussion points are meant to aid the session discussion. Facilitators can adjust the talking points according to their needs and the flow of the session.

**Educational objectives:**

1. Describe the main elements of a research project.
2. Critically appraise the main elements of a research project.
3. Create action steps in SMART format.
4. Discuss the iterative nature of designing a research project.

**Session Plan**

Total session time: 90 min

***Part 1. Introduction (15 minutes)***

The session starts with the main facilitator explaining the session’s objectives, methodology, and timeline. We recommend explaining the Sailboat activity timeline, reviewing the elements of the Sailboat as a general representation of a Health Profession Education Research (HPER) project, providing ideas for the small group project consultation, and explaining the development of the action plan in SMART format.

Discussion points for the large group introduction:

1. The Sailboat represents the participants’ HPER project idea. Before the session, each participant created a Sailboat that presents their project idea. Participants used the following characteristics to describe their project idea **(Educational Objective 1).**

- Body of the boat: Research Question
- Sails: Research Problem and Conditions
- Windows: Variables, Population, Sample, Outcome measures
- Flag: Research Design
- Island: The final goal or significance of your project
- Water around the island and the sailboat: research site
- Iceberg, which represents the major problem that might arise during project implementation. This is something that can ruin the HPER project.

Ask participants to explain their project idea to their partner using the Sailboats as a visual representation of their HPER idea, when they are in the breakout room.

1. Participants were also asked to use the wind and the anchors on their Sailboat to represent the strengths and weaknesses of the project. **(Educational Objective 2)**

Remind participants to describe the strengths and weaknesses of their project with their partner in the breakout room discussion. Encourage participants to find solutions for the weaknesses, consolidate them if possible. Use the strengths of the project to mitigate the challenges.

1. While in the breakout room, ask participants to create an action plan using the SMART format. **(Educational Objective 3)**

*S = Specific – Is the action plan focused on a clear aim?*

*M = Measurable: Could someone determine whether you have completed your action plan?*

*A = Achievable: Do you have the resources and capabilities to complete your action plan?*

*R = Relevant: Does the action plan align with the challenge/weakness you identified?*

*T = Time-bound: Does the action plan have a deadline?*

*Discussion points for the small group project consultation:*

*Encourage participants to discuss the questions below for each HPER project in their breakout room.*

1. *How can you characterize your HPER project?* ***(Educational Objective 1)***

*Please discuss your HPER project’s relevance, interest, originality, clarity, feasibility, answerability, and generalizability.*

*Use the PICOTS (Population, Intervention, Control, Outcomes, Time, Setting), I-SMART (Important, Specific, Measurable, Achievable, Relevant, Timely) or FINER (Feasible, Important and Interesting, Ethical, Relevant) criteria to evaluate the completeness and strength of your research question(s).* ***(Educational Objective 1)***

1. *Did you use a theoretical or conceptual framework to situate the HPER project?*

*A clear conceptual framework includes relevant educational theories that may justify the research design, data collection and analysis plan. In addition, the conceptual framework provides insights into how the project’s findings might be generalizable to other educational contexts.* ***(Educational Objective 1)***

1. *Did you complete a literature search to support the prior work and the gaps in knowledge?* ***(Educational Objective 1)***

*A thorough review of the literature can help the researcher in planning a HPER project in several ways. For example: A) understanding what is known and unknown about a research problem, B) providing guidance on how well the research question can answer the gaps in knowledge, C) estimating the importance and relevance of the HPER project for the larger medical education field.*

1. *What are the strengths and weaknesses of your project plan?* ***(Educational Objective 2)***

*Does the research design align with the research question? Using the proposed research design will you (the project owner) be able to answer the research question? Keep in mind that the research question directs the selection of the research design. Common research designs include 1) Exploratory (goal: by qualitative or quantitative data collection these studies aim to explain a phenomenon, situation or relationships), 2) Experimental (goal: by assigning individuals to different conditions, these studies aim to justify the use of an intervention), 4) Observational (goal: by examining natural groups of individuals, these studies aim to predict a selected set of outcomes), 5) Translational (goal: by collecting data from complex clinical settings, or from the synthesis of the literature, these studied aim to provide guidance on knowledge implementation), and 6) Mixed-methods research designs (goal: by integrating qualitative and quantitative data, these studies aim to provide* a more complete analysis of educational practices, outcomes, and experiences)*.*

*The outcome measures can be assessed through the Kirkpatrick’s evaluation model, which has four levels of educational outcomes.*

*Level 1= reaction (learner satisfaction)*

*Level 2: learning (knowledge, skills, and attitudes),*

*Level 3: behavior change*

*Level 4: patient impact*

*HPEs are encouraged to aim high level outcome measures on the Kirkpatrick’s pyramid*

1. *Is the project feasible? Do you have a research team? Are you ready to implement the research plan?* ***(Educational Objective 2)***

*Discuss with your partner if you have the resources (e.g.; data collection, management, analysis, literature search access, citation manager), team members, institutional support, access to learners to complete the project.*

1. *How can we mitigate the project’s challenges? Together with your partner craft a SMART action plan to address one or more of the project weaknesses/challenges you identified. Once you crafted your action plan, please test it against the SMART criteria listed below****. (Educational Objective 3)***

*S = Specific – Is the action plan focused on a clear aim?*

*M = Measurable: Could someone determine whether you have completed your action plan?*

*A = Achievable: Do you have the resources and capabilities to complete your action plan?*

*R = Relevant: Does the action plan align with the challenge/weakness you identified?*

*T = Time-bound: Does the action plan have a deadline?*

In the meantime, the second facilitator creates the breakout rooms (two participants per breakout room). The second facilitator adds the link to the chat to the Sailboat collaborative working area (if used) remind participants to click on the link to access their Sailboats. If the facilitators decide not to use a collaborative platform, then they should remind participants to share their Sailboats in the breakout room using the share screen function of the videoconferencing platform.

***Part 2. Breakout room: small group discussion (40 minutes)***

We recommend that facilitators follow up on participants' work. They can visit the breakout rooms and answer questions and provide guidance if needed. Additionally, they should turn on the timer and set it for 13 minutes for the project discussion, followed by 7 minutes for working on the action plan. This timing should be applied to each participant.

***Part 3. Group Presentation (20 minutes)***

When the participants come back from the breakout rooms, the lead facilitator asks for two volunteers to present their projects. Each presenter will have 10 minutes, where the participant will summarize the project, briefly explain its strengths (wind), weaknesses or challenges (anchors and iceberg), and describe the SMART action steps they came up with during the small group discussion. The other participants and facilitators can suggest ideas for addressing the project’s weaknesses and action steps.

Discussion points for the large group discussion. **(Educational Objectives 1-3)**

To reduce cognitive load and stay on time, we recommend pasting the following instructions into the chat:

For the presenter, please focus on:

- A brief summary of the project
- Project’s strengths and weakness that you identified in your small group discussion
- Present your SMART action steps

For the group, please think about these questions:

- What do you think of the weaknesses of the project?
- What do you think of the SMART action steps?

***Part 4. Reflection (15 minutes)***

This final part of the session aims to foster participants’ metacognition about their views about HPER project planning, experience with the activity, and its impact on their project development **(Educational Objective 4).**

We recommend starting with a personal reflection on the session’s takeaway and the process of planning a HPER project.

Discussion points for the large group discussion:

A useful prompt for this aim is one of the Project’s Zero Thinking Routines: “I used to think… Now I think...”. You can offer participants two minutes to reflect and ask them to write their answers in the chat. However, they should submit their responses (hit ‘send’) only after the time is over, to allow everyone time to think without the distraction of the chat. This approach is known as ‘chat waterfall’ since participants’ comments appear all at once, resembling the flow of a waterfall.

Finally, facilitators can end the session with a group reflection on participants’ answers to the prompt and insights about their experience with the *Sailboat activity*.

In the last five minutes of the workshop, we asked participants to complete our post-workshop survey (Appendix G). We recommend using this (or a similar) survey to collect participants’ feedback on the Sailboat activity.
