# Supplementary material for: The Sailboat Activity: An Interactive, Visually Engaging Approach to Design and Assess Health Profession Education Research Projects
Source: MedEdPORTAL. 2025 May 2;21:11520. doi: 10.15766/mep_2374-8265.11520 (PMC12046060; doi:10.15766/mep_2374-8265.11520)
Supplement: Supplementary file 1 — Sailboat Template.pptxPreworkshop Assignment Instructions.docxPreworkshop Survey.docxFacilitator Guide.docxSailboat Activity Session Slides.pptxCollaborative Working Area.pptxPostworkshop Survey.docxAction Plan Scoring Rubric.docx [file mep_2374-8265.11520-s001.zip › E. Sailboat Activity Session Slides.pptx]

## Slide 1
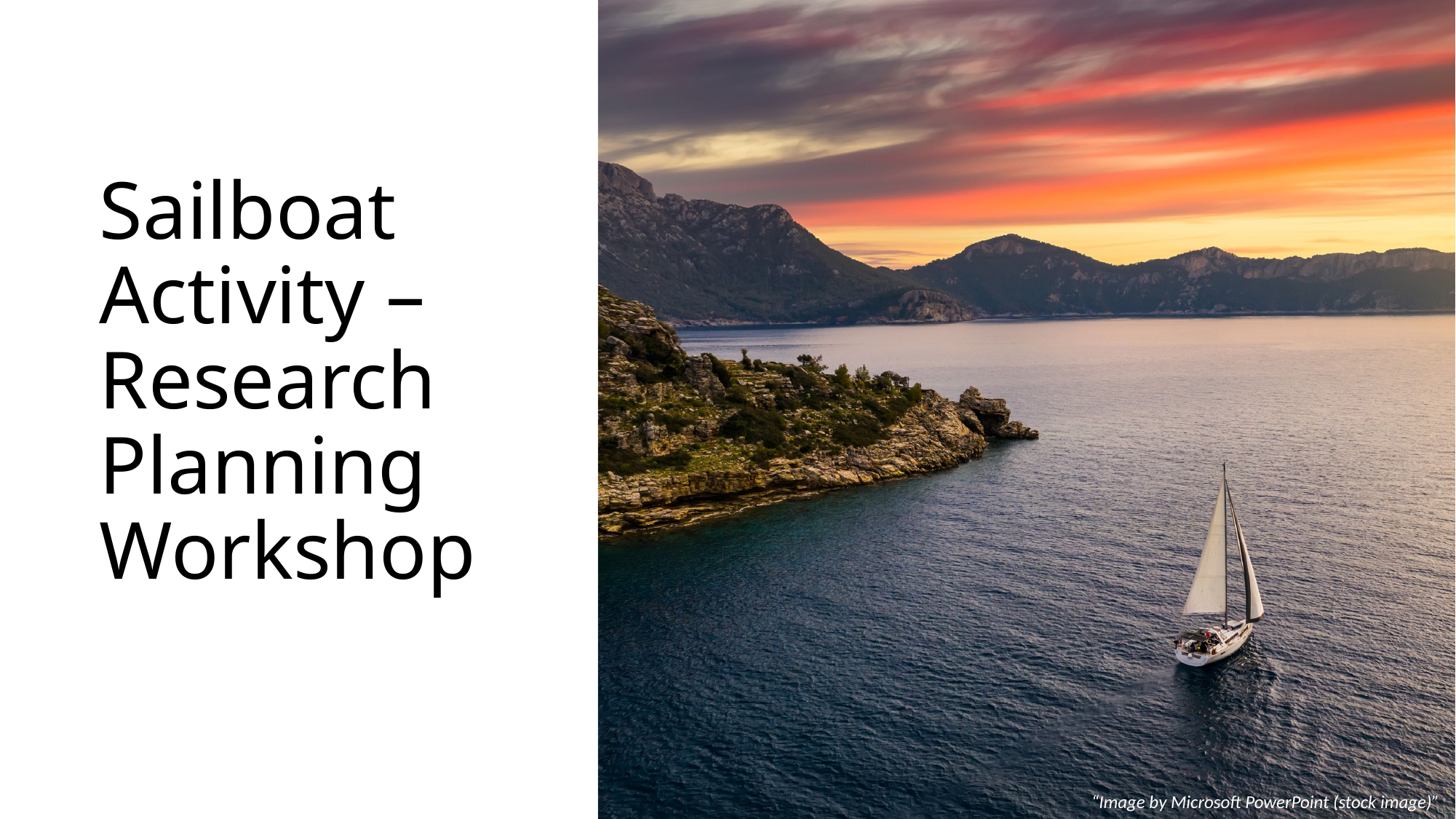

# Sailboat Activity – Research Planning Workshop
“Image by Microsoft PowerPoint (stock image)”

## Slide 2
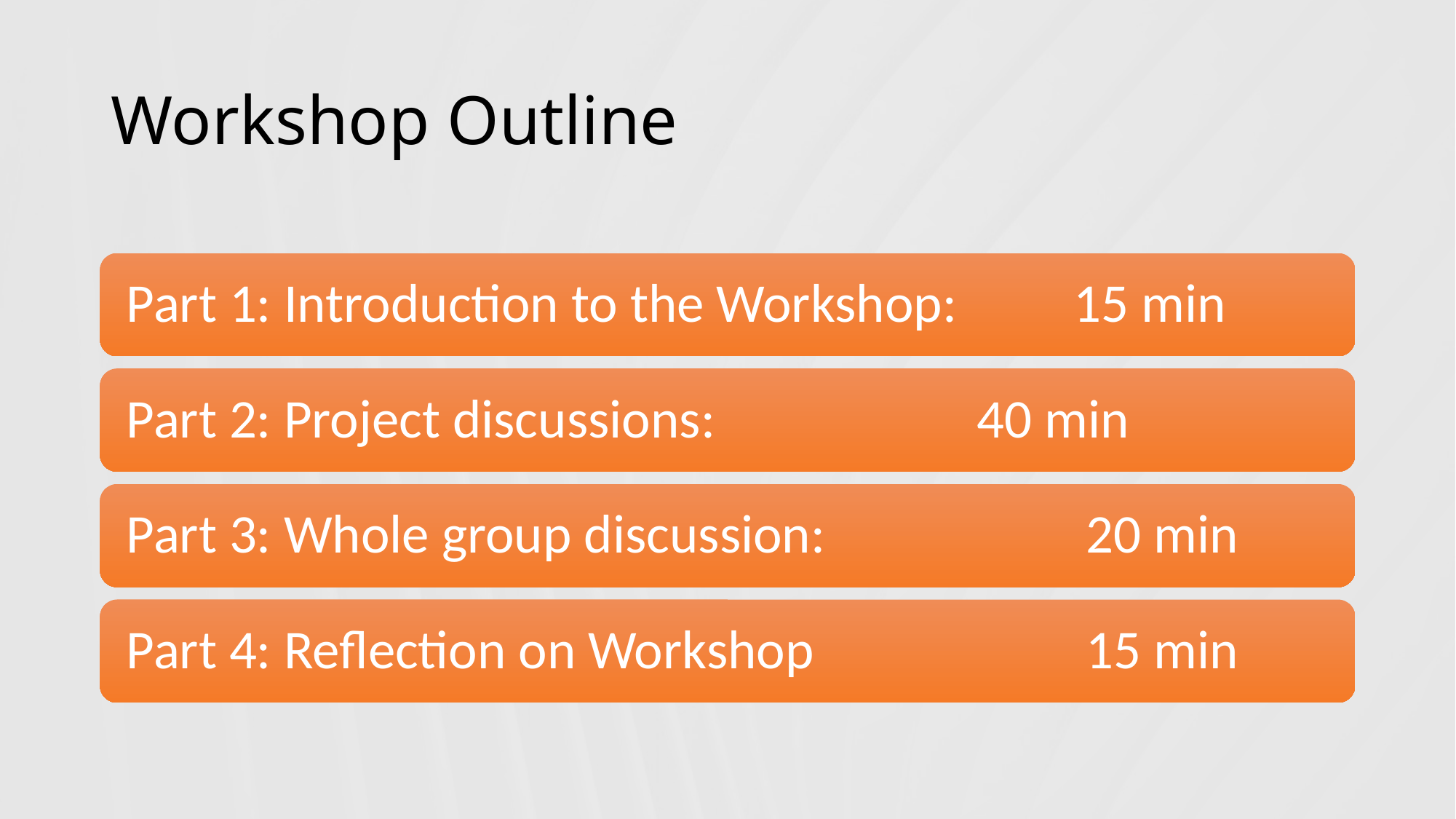

# Workshop Outline

## Slide 3
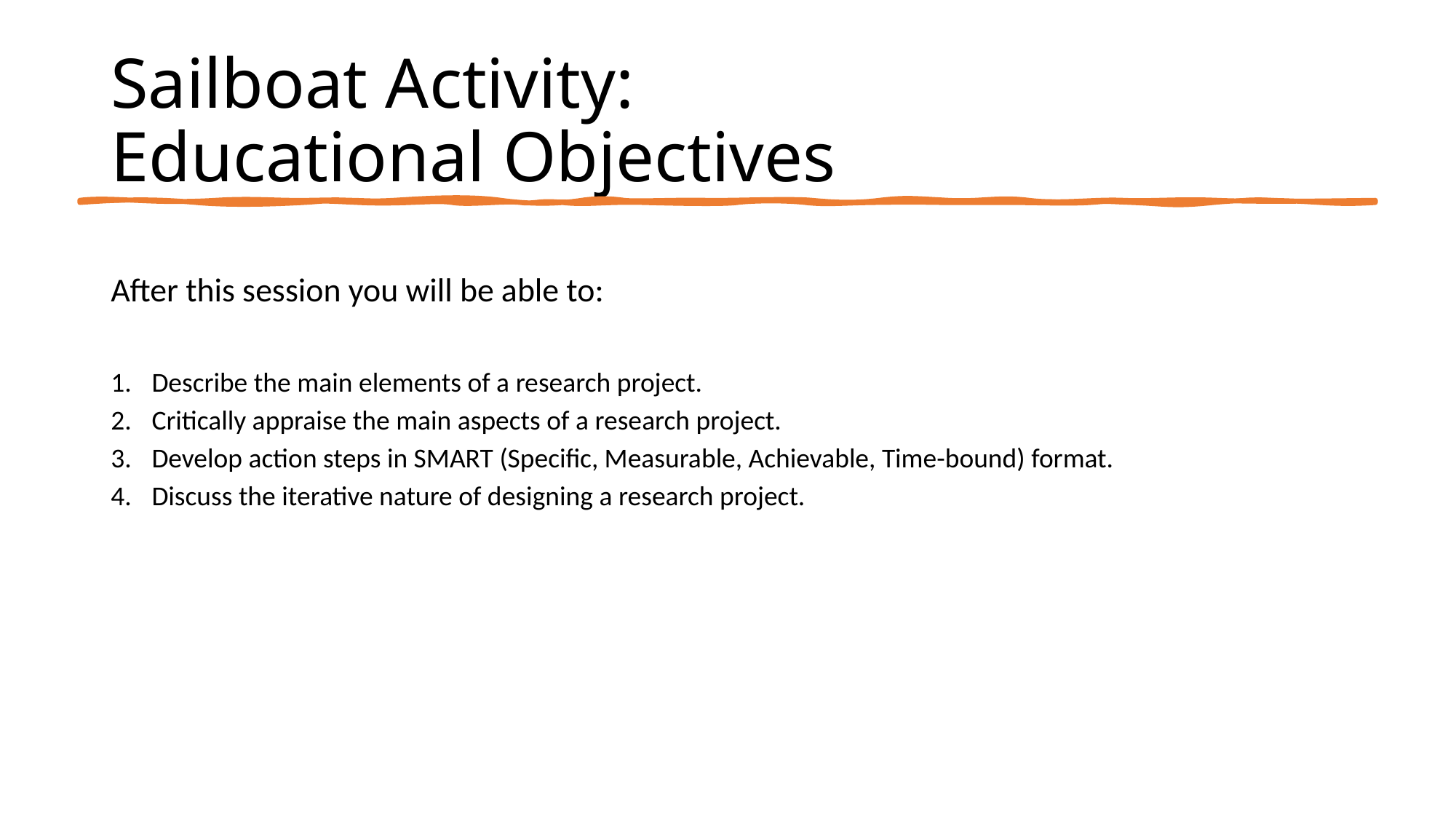

# Sailboat Activity: Educational Objectives
After this session you will be able to:
Describe the main elements of a research project.
Critically appraise the main aspects of a research project.
Develop action steps in SMART (Specific, Measurable, Achievable, Time-bound) format.
Discuss the iterative nature of designing a research project.

## Slide 4
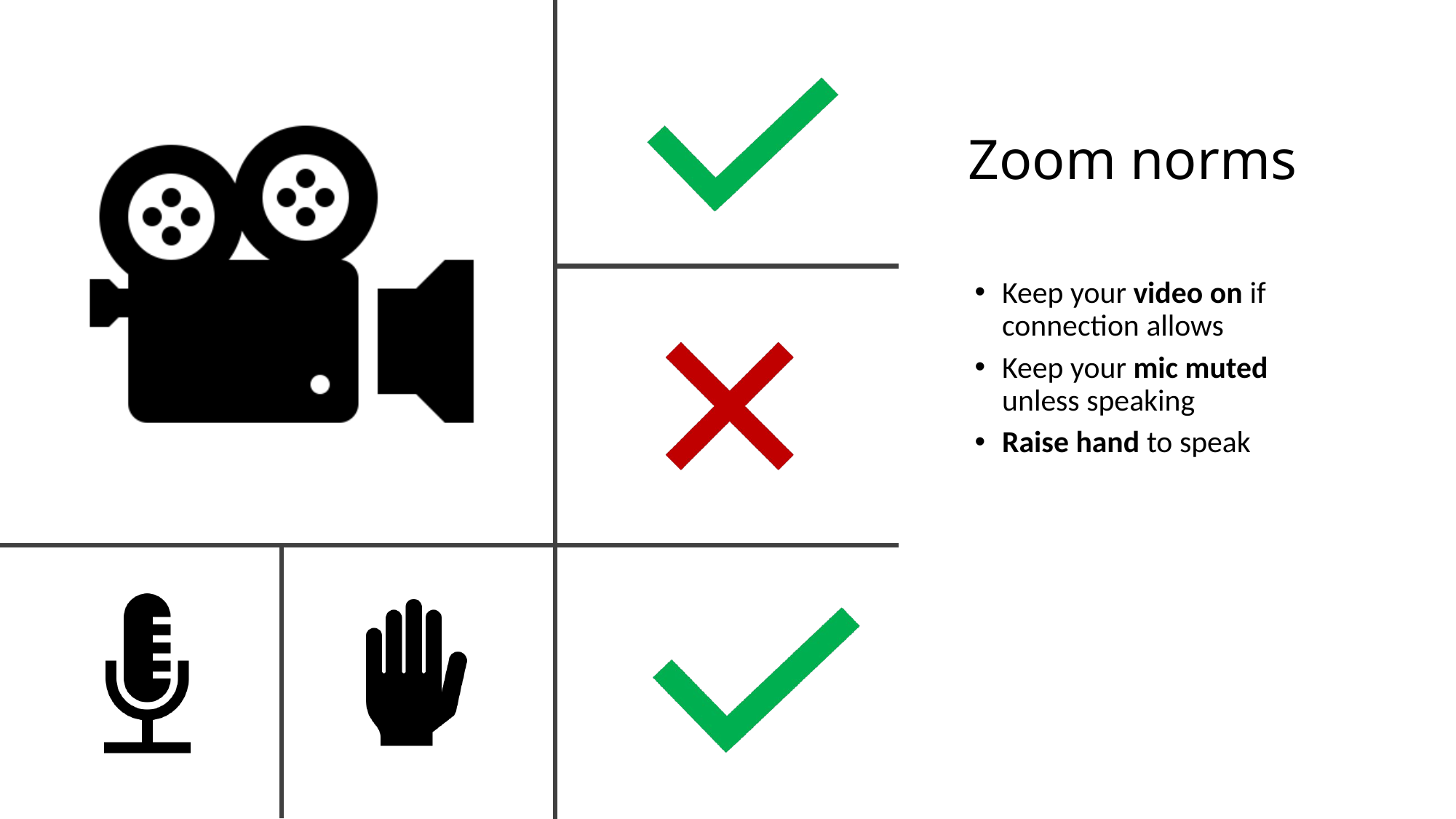

# Zoom norms
Keep your video on if connection allows
Keep your mic muted unless speaking
Raise hand to speak

## Slide 5
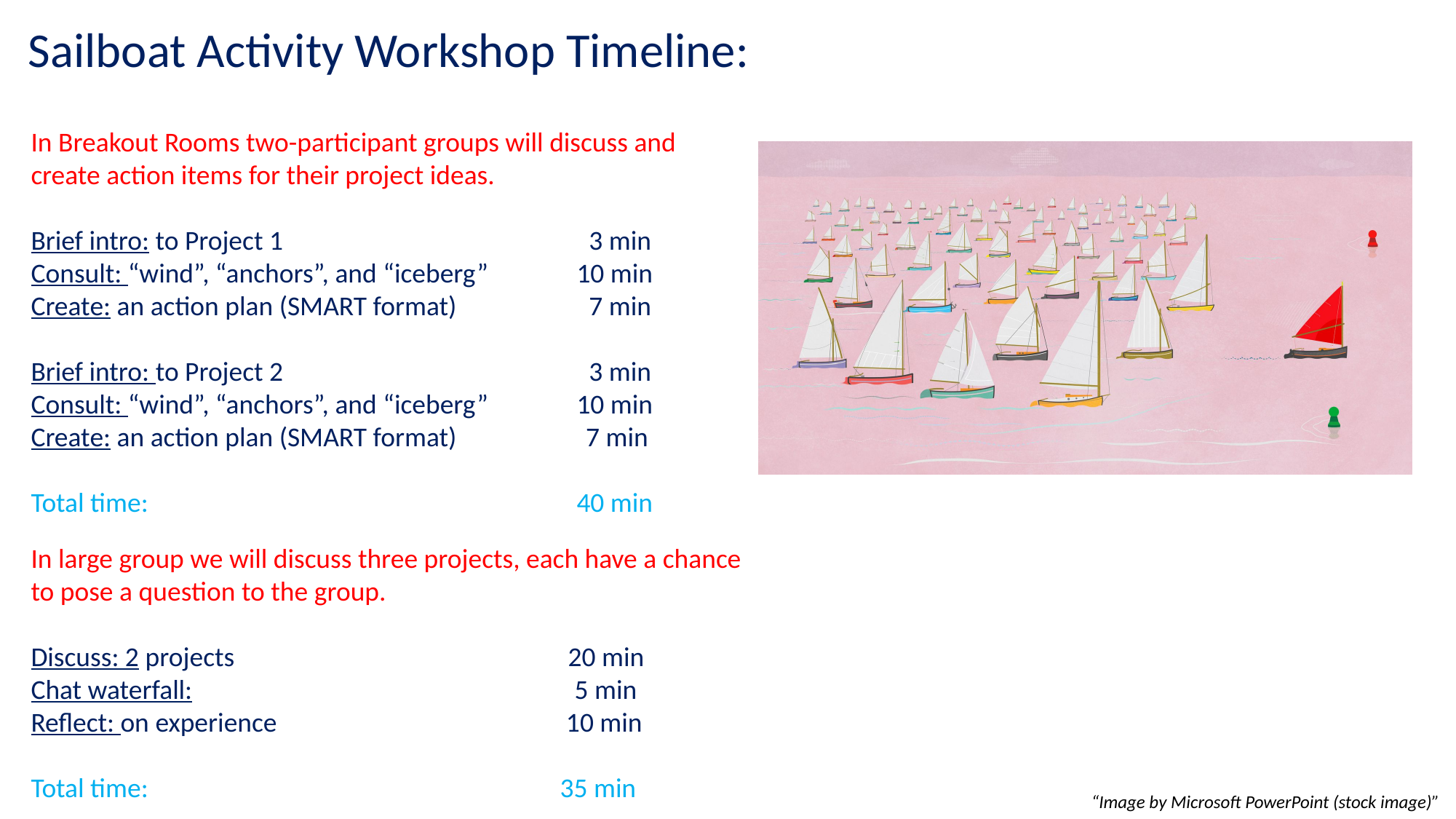

Sailboat Activity Workshop Timeline:
In Breakout Rooms two-participant groups will discuss and create action items for their project ideas.
Brief intro: to Project 1			 3 min
Consult: “wind”, “anchors”, and “iceberg” 	10 min
Create: an action plan (SMART format) 		 7 min
Brief intro: to Project 2 			 3 min
Consult: “wind”, “anchors”, and “iceberg” 	10 min
Create: an action plan (SMART format) 7 min
Total time:				40 min
In large group we will discuss three projects, each have a chance to pose a question to the group.
Discuss: 2 projects 		 20 min
Chat waterfall: 5 min
Reflect: on experience		 10 min
Total time:			 35 min
“Image by Microsoft PowerPoint (stock image)”

## Slide 6
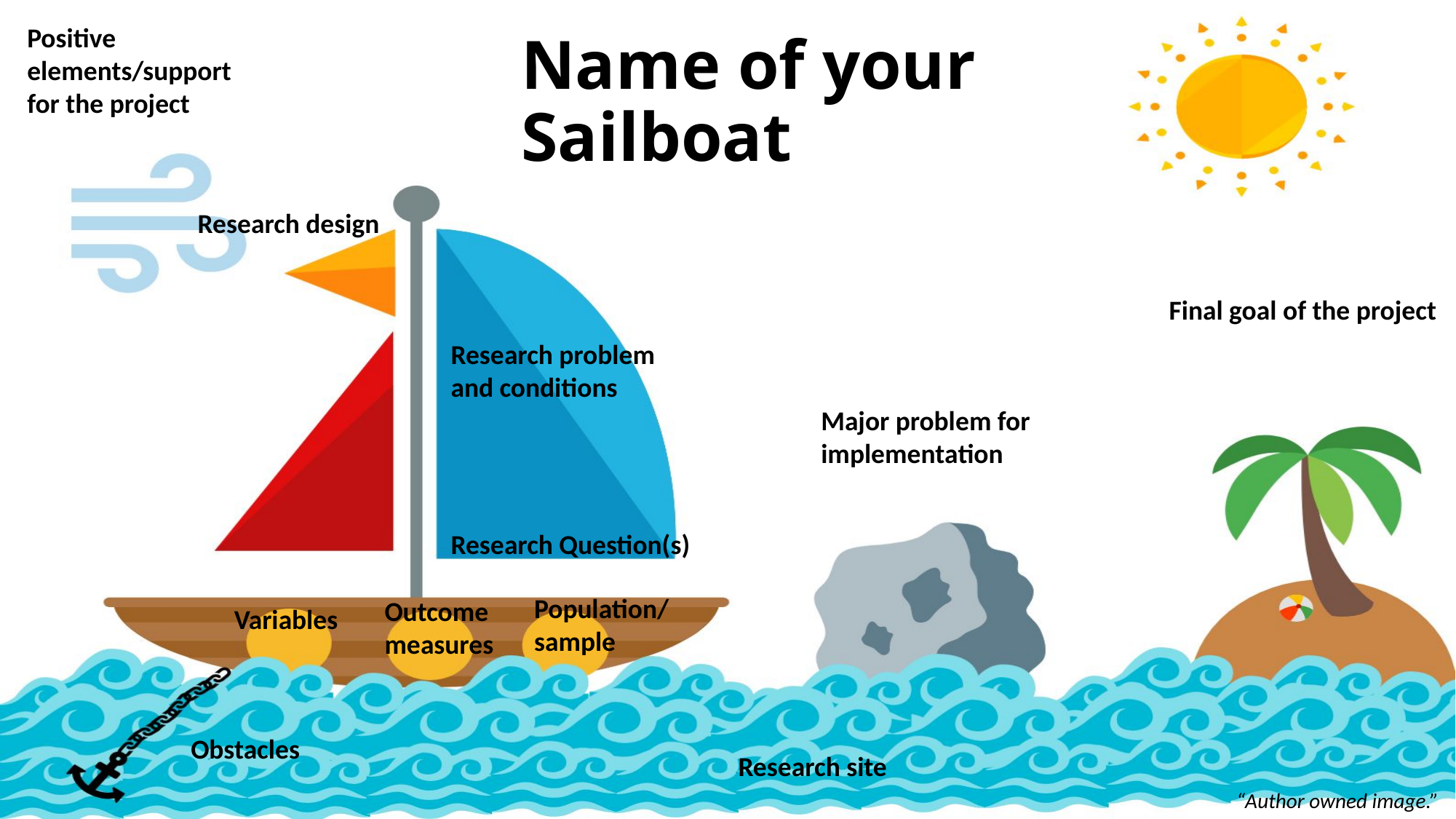

Positive elements/support for the project
Name of your Sailboat
Research design
Final goal of the project
Research problem and conditions
Major problem for implementation
Research Question(s)
Population/ sample
Outcome measures
Variables
Obstacles
Research site
“Author owned image.”

## Slide 7
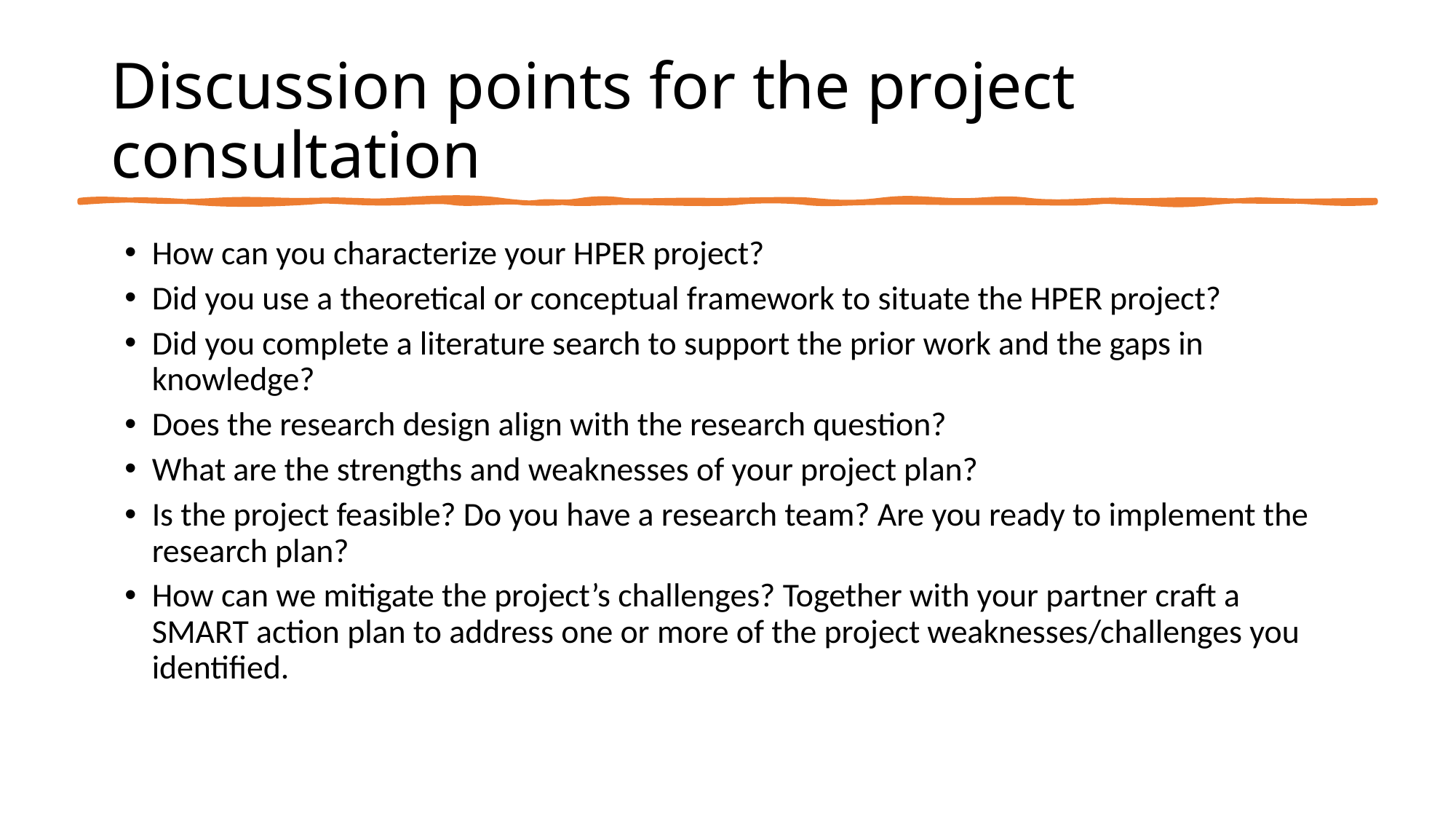

# Discussion points for the project consultation
How can you characterize your HPER project?
Did you use a theoretical or conceptual framework to situate the HPER project?
Did you complete a literature search to support the prior work and the gaps in knowledge?
Does the research design align with the research question?
What are the strengths and weaknesses of your project plan?
Is the project feasible? Do you have a research team? Are you ready to implement the research plan?
How can we mitigate the project’s challenges? Together with your partner craft a SMART action plan to address one or more of the project weaknesses/challenges you identified.

## Slide 8
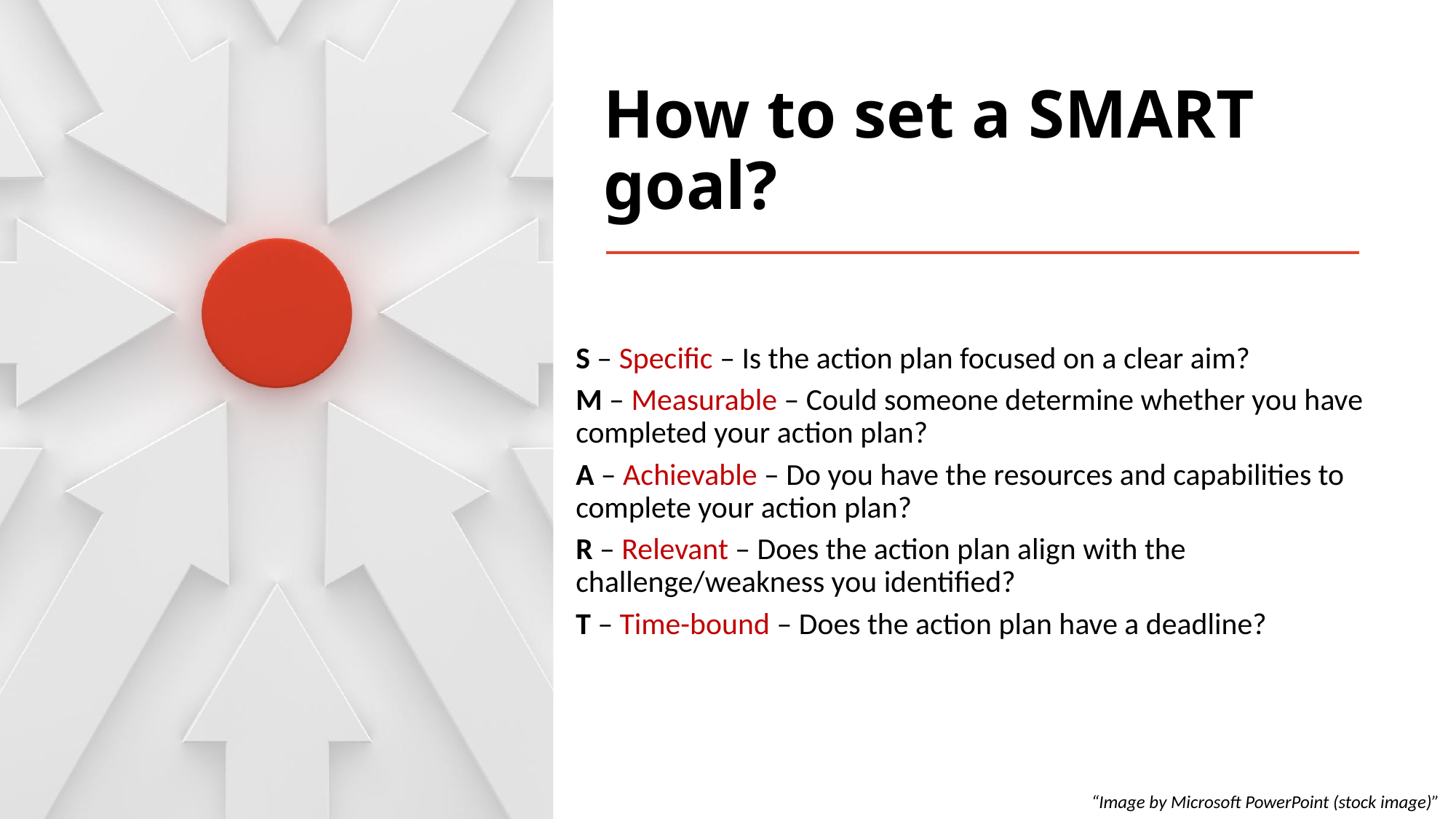

How to set a SMART goal?
S – Specific – Is the action plan focused on a clear aim?
M – Measurable – Could someone determine whether you have completed your action plan?
A – Achievable – Do you have the resources and capabilities to complete your action plan?
R – Relevant – Does the action plan align with the challenge/weakness you identified?
T – Time-bound – Does the action plan have a deadline?
“Image by Microsoft PowerPoint (stock image)”

## Slide 9
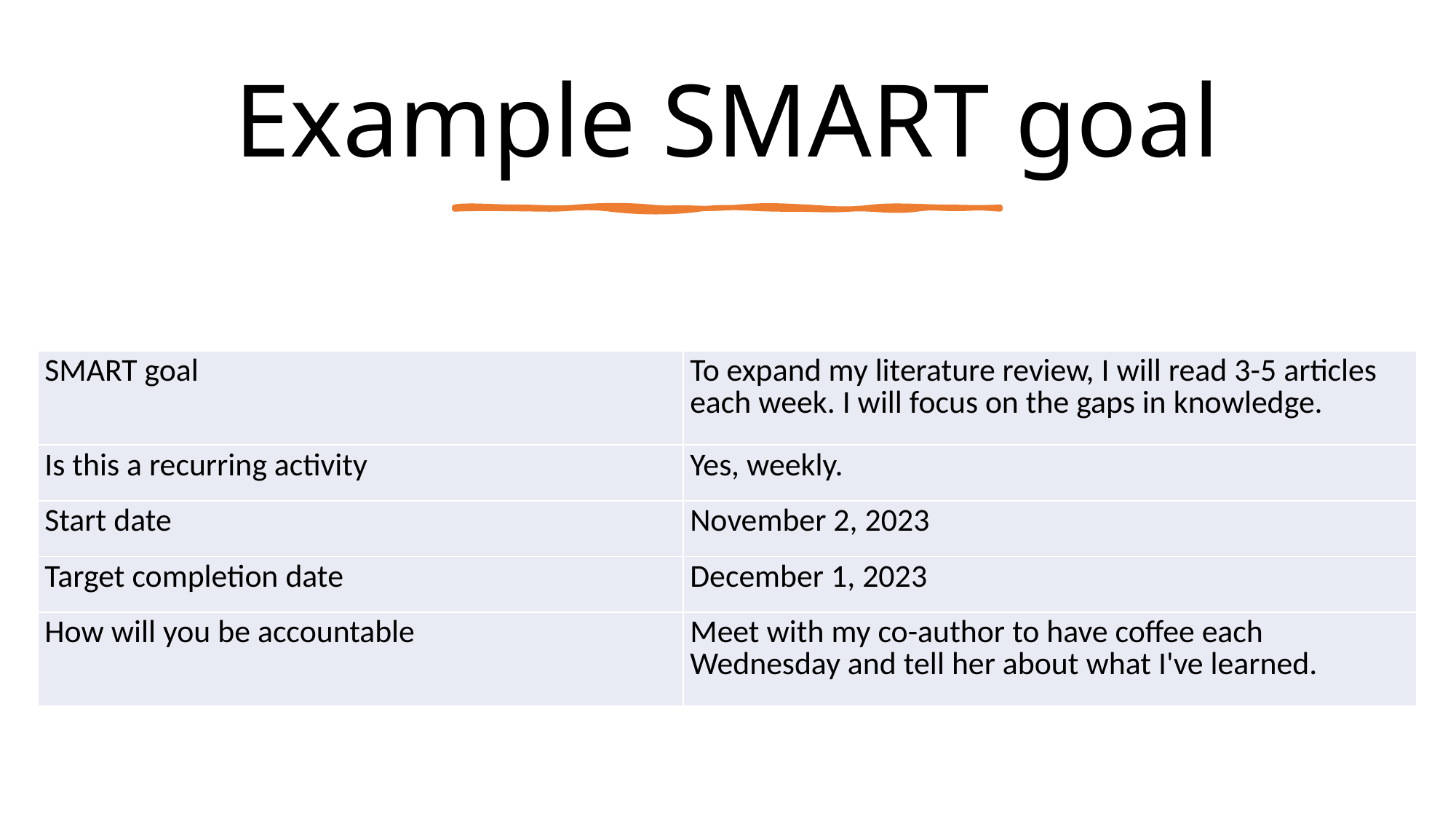

# Example SMART goal
| SMART goal | To expand my literature review, I will read 3-5 articles each week. I will focus on the gaps in knowledge. |
| --- | --- |
| Is this a recurring activity | Yes, weekly. |
| Start date | November 2, 2023 |
| Target completion date | December 1, 2023 |
| How will you be accountable | Meet with my co-author to have coffee each Wednesday and tell her about what I've learned. |

## Slide 10
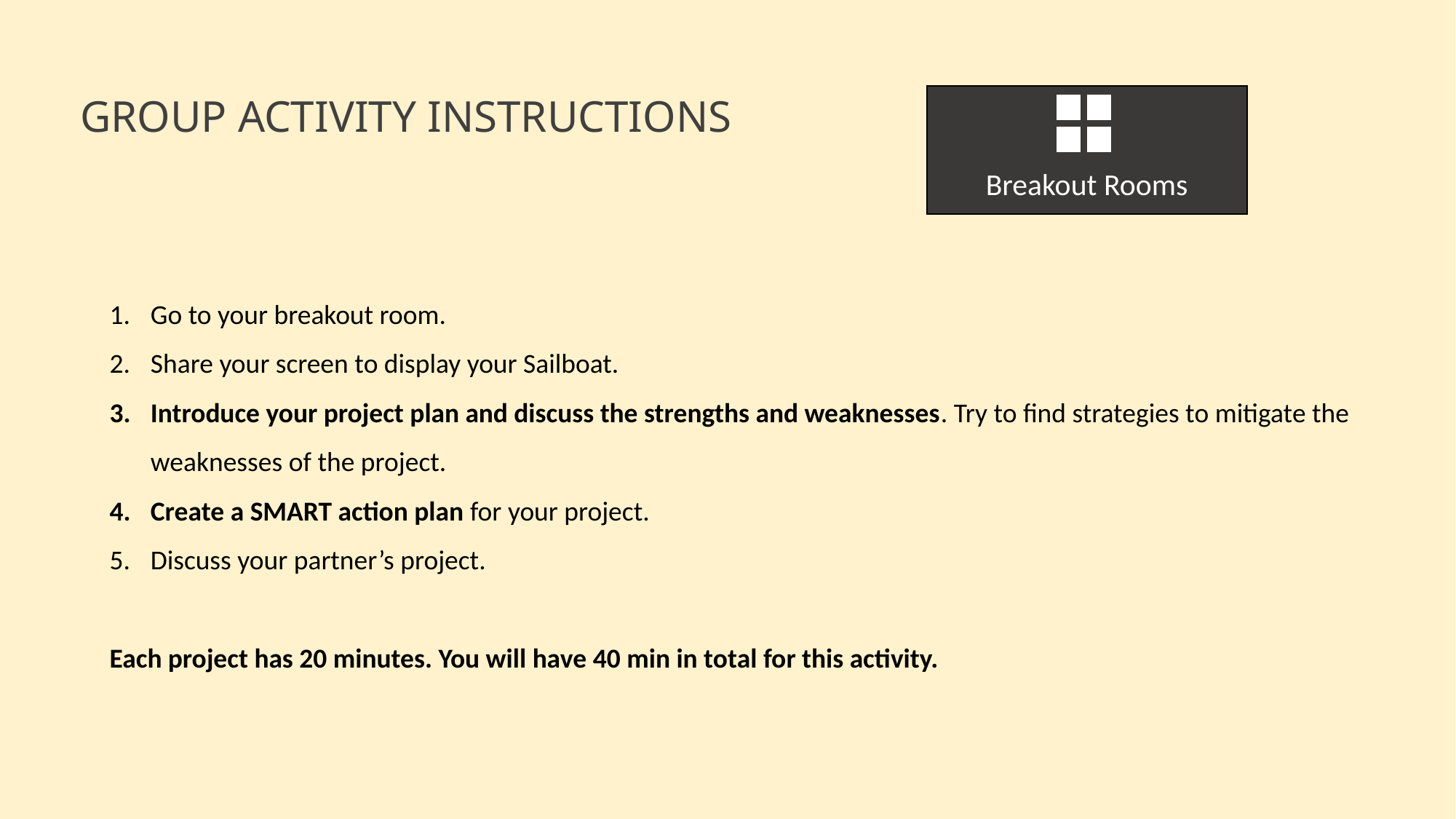

Group activity instructions
Breakout Rooms
Go to your breakout room.
Share your screen to display your Sailboat.
Introduce your project plan and discuss the strengths and weaknesses. Try to find strategies to mitigate the weaknesses of the project.
Create a SMART action plan for your project.
Discuss your partner’s project.
Each project has 20 minutes. You will have 40 min in total for this activity.

## Slide 11
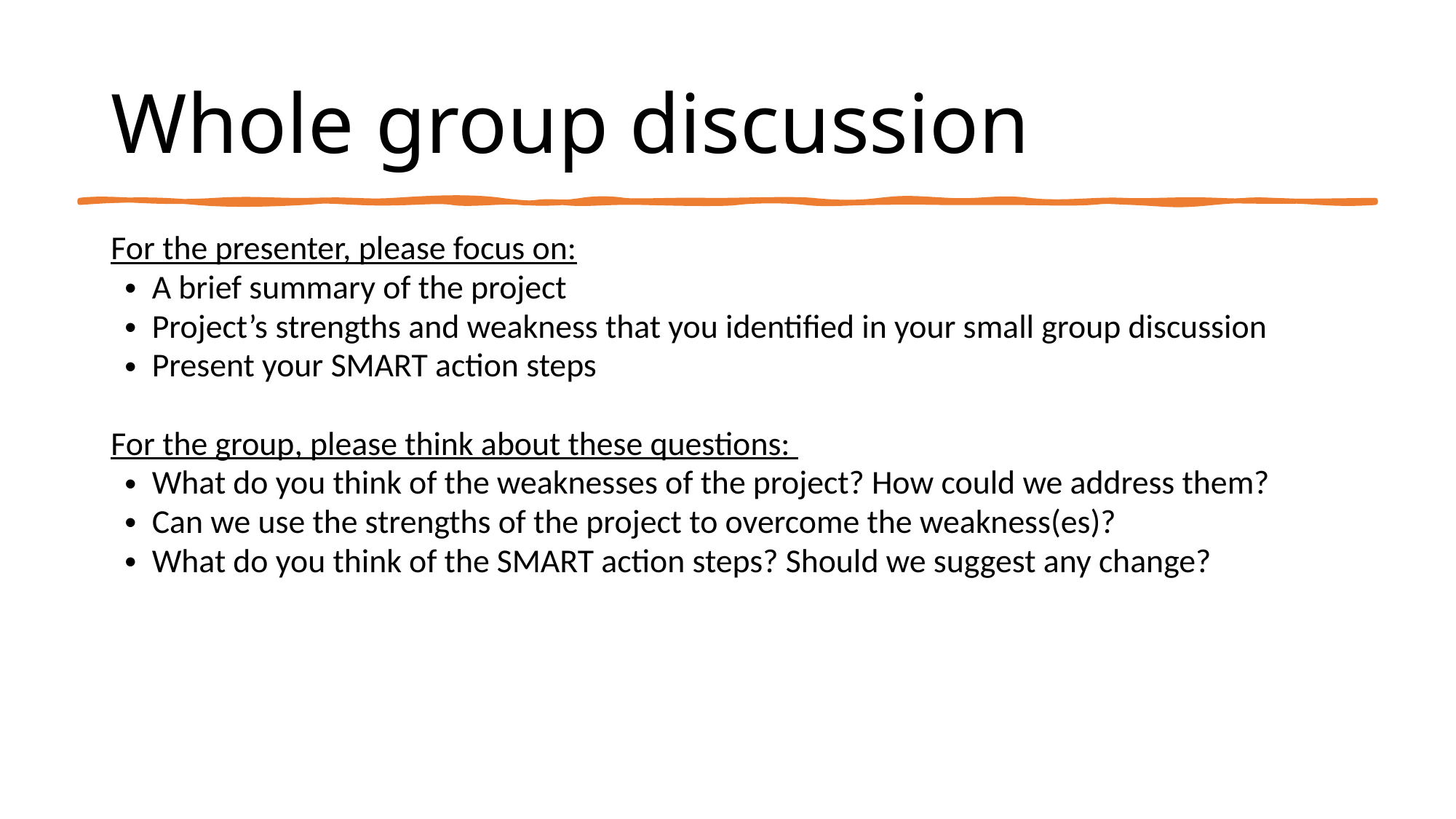

# Whole group discussion
For the presenter, please focus on:
A brief summary of the project
Project’s strengths and weakness that you identified in your small group discussion
Present your SMART action steps
For the group, please think about these questions:
What do you think of the weaknesses of the project? How could we address them?
Can we use the strengths of the project to overcome the weakness(es)?
What do you think of the SMART action steps? Should we suggest any change?

## Slide 12
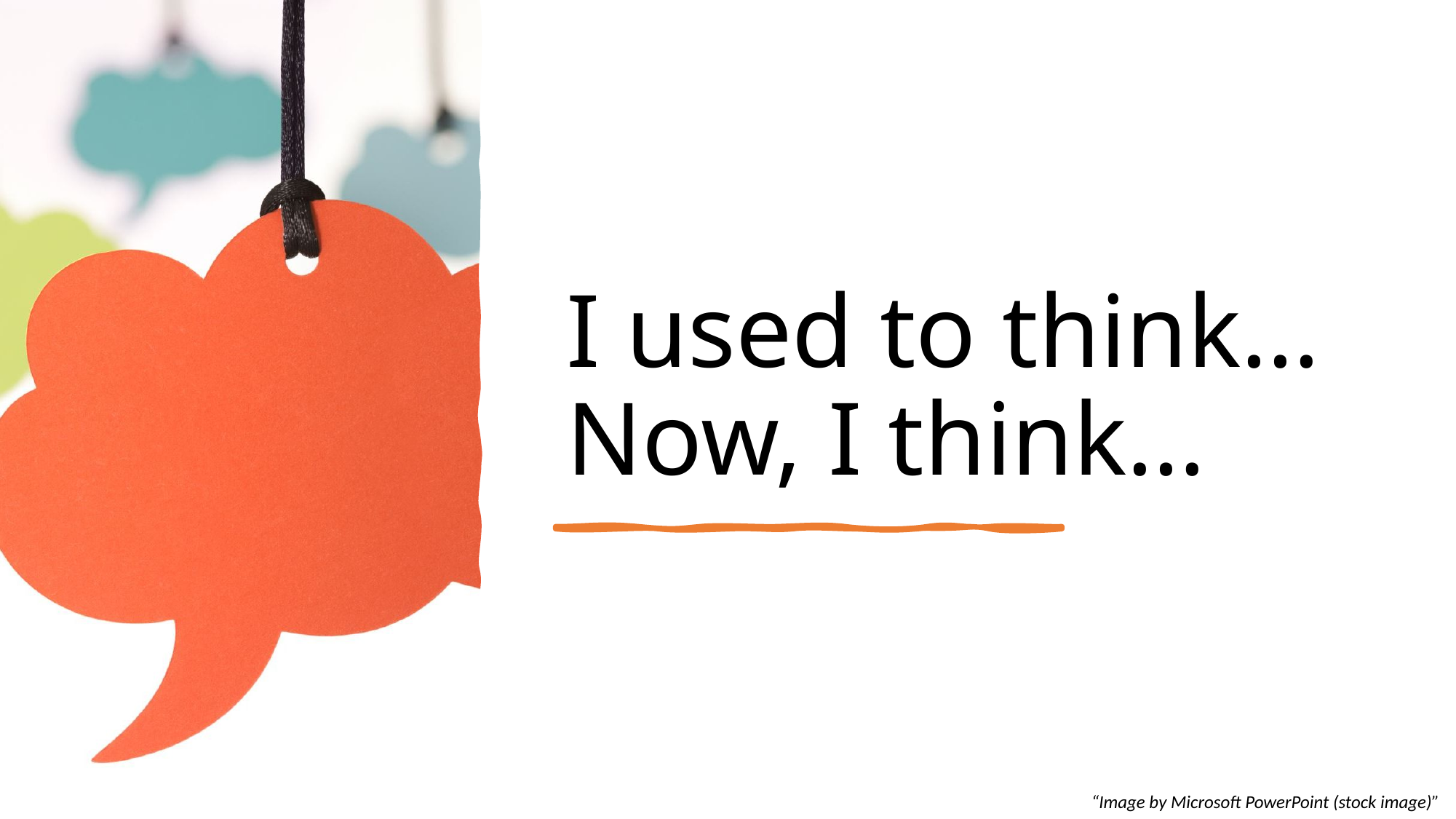

# I used to think… Now, I think…
“Image by Microsoft PowerPoint (stock image)”

## Slide 13
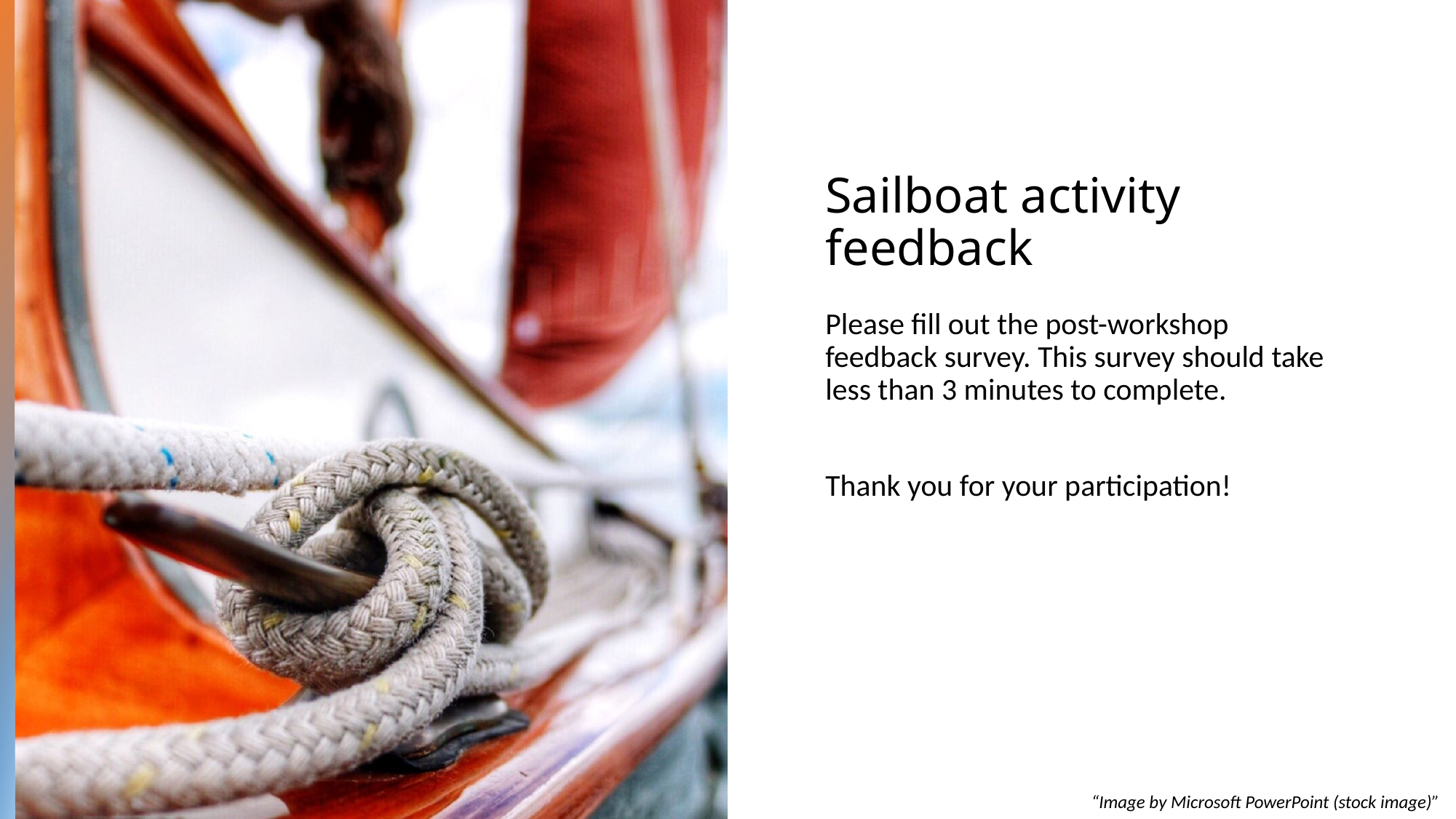

# Sailboat activity feedback
Please fill out the post-workshop feedback survey. This survey should take less than 3 minutes to complete.
Thank you for your participation!
“Image by Microsoft PowerPoint (stock image)”

## Slide 14
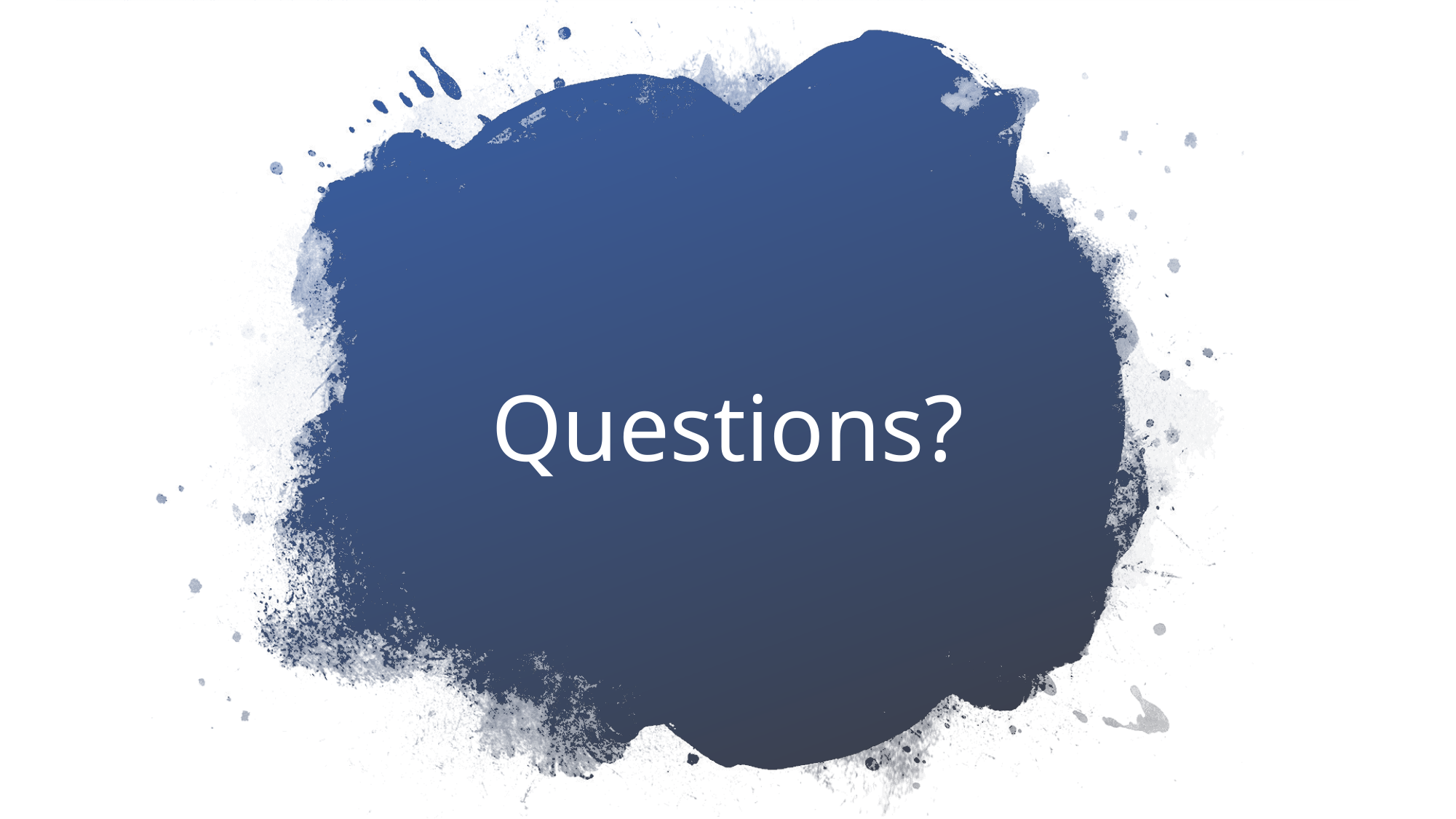

# Questions?
