# Supplementary material for: The Sailboat Activity: An Interactive, Visually Engaging Approach to Design and Assess Health Profession Education Research Projects
Source: MedEdPORTAL. 2025 May 2;21:11520. doi: 10.15766/mep_2374-8265.11520 (PMC12046060; doi:10.15766/mep_2374-8265.11520)
Supplement: Supplementary file 1 — Sailboat Template.pptxPreworkshop Assignment Instructions.docxPreworkshop Survey.docxFacilitator Guide.docxSailboat Activity Session Slides.pptxCollaborative Working Area.pptxPostworkshop Survey.docxAction Plan Scoring Rubric.docx [file mep_2374-8265.11520-s001.zip › F. Collaborative Working Area.pptx]

## Slide 1
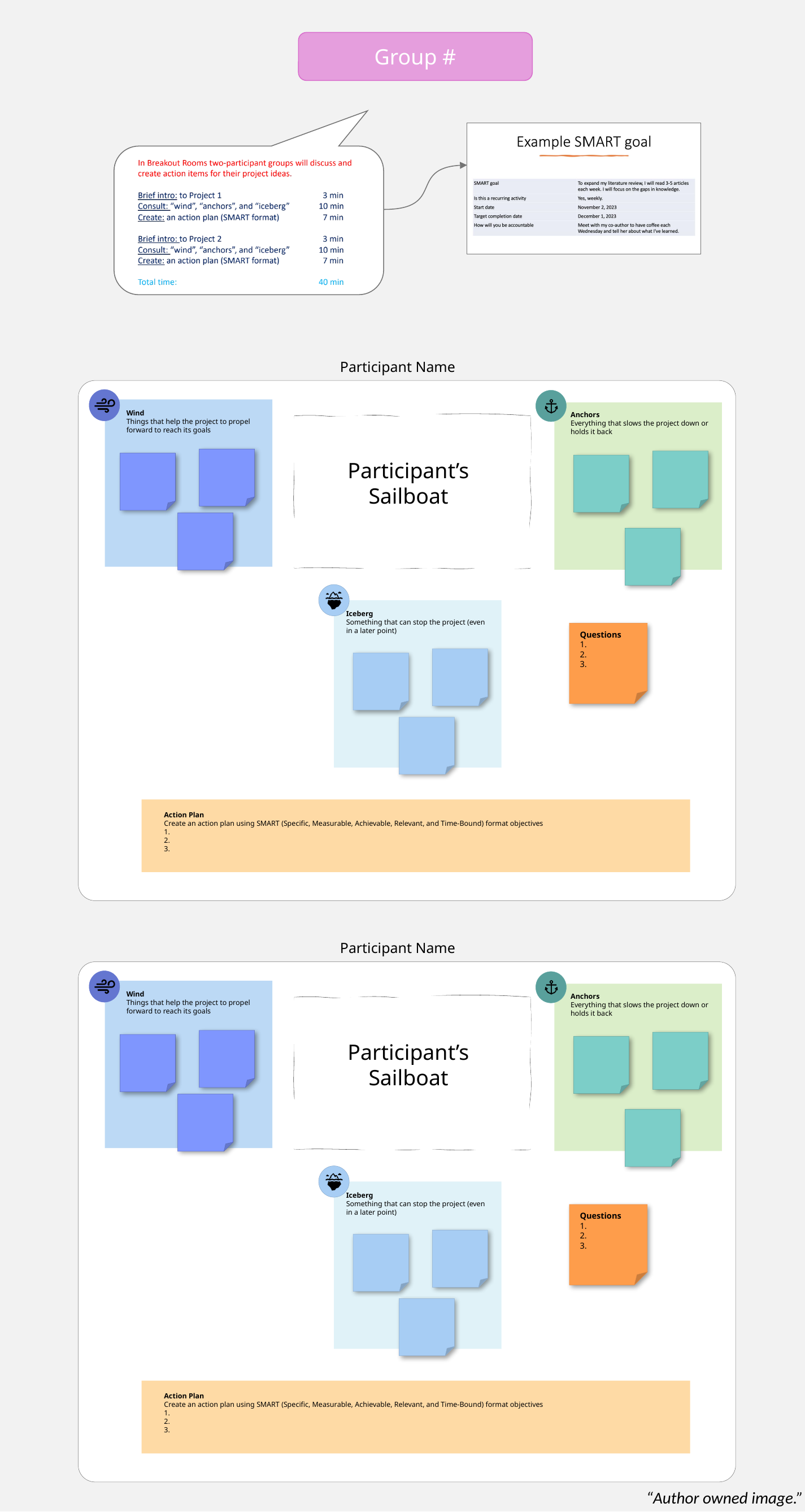

Group #
Participant Name
Wind
Things that help the project to propel forward to reach its goals
Anchors
Everything that slows the project down or holds it back
Participant’s Sailboat
Iceberg
Something that can stop the project (even in a later point)
Questions
1.
2.
3.
Action Plan
Create an action plan using SMART (Specific, Measurable, Achievable, Relevant, and Time-Bound) format objectives
1.
2.
3.
Participant Name
Wind
Things that help the project to propel forward to reach its goals
Anchors
Everything that slows the project down or holds it back
Participant’s Sailboat
Iceberg
Something that can stop the project (even in a later point)
Questions
1.
2.
3.
Action Plan
Create an action plan using SMART (Specific, Measurable, Achievable, Relevant, and Time-Bound) format objectives
1.
2.
3.
“Author owned image.”
