# Supplementary material for: The Sailboat Activity: An Interactive, Visually Engaging Approach to Design and Assess Health Profession Education Research Projects
Source: MedEdPORTAL. 2025 May 2;21:11520. doi: 10.15766/mep_2374-8265.11520 (PMC12046060; doi:10.15766/mep_2374-8265.11520)
Supplement: Supplementary file 1 — Sailboat Template.pptxPreworkshop Assignment Instructions.docxPreworkshop Survey.docxFacilitator Guide.docxSailboat Activity Session Slides.pptxCollaborative Working Area.pptxPostworkshop Survey.docxAction Plan Scoring Rubric.docx [file mep_2374-8265.11520-s001.zip › _Educational Summary Report_11520.pdf]

MedEdPORTAL® The AAMC Journal of Teaching and Learning Resources

Original Publication Open Access

The Sailboat Activity: An Interactive, Visually Engaging Approach to Design and Assess Health Profession Education Research Projects

Dominique Harz, DMD, MDS, MMSc, Jennifer Kesselheim, MD, MEd, Krisztina Fischer, MD, PhD, MMSc*

*Corresponding author: krisztina_fischer@hms.harvard.edu

Abstract

Introduction: Limited guidance exists to engage health profession educators in designing and critically evaluating rigorous research projects. Traditional lectures are often insufficient for advancing educators' skills. By leveraging educational theories, we designed and implemented an interactive, learner-focused, reflective workshop—the Sailboat activity—offering educators an opportunity to evaluate their research plans and hone their research planning skills. **Methods:** Preworkshop, participants represented their research plan on a Sailboat diagram and completed a preworkshop survey assessing their confidence in designing a research project. During the 90-minute virtual workshop, participants discussed their projects in pairs, and developed Specific, Measurable, Achievable, Relevant, Time-bound action plans. The group then reconvened to review two projects and reflect on the activity. Finally, participants completed a postworkshop survey. **Results:** Thirty-four educators from 17 diverse specialties participated in the 2021, 2022, and 2023 Sailboat workshops. Pre- and postworkshop survey response rates were high (>90%). Participants indicated that they found the workshop valuable, highlighting that it helped them refine their project plans and organize their thoughts while fostering peer interaction. Responses to open-ended questions revealed that the workshop enhanced participants' understanding of the importance of iterative research planning. We observed significant increases from pre- to postworkshop ($p < .05$) in participants' confidence regarding identifying project barriers, weaknesses, key elements, alignment, and subsequent steps. **Discussion:** The Sailboat activity is an effective, feasible opportunity for educators to critically appraise and refine Health Professions Education Research projects, even in virtual environments. It could be adapted for other academic environments and incorporated into existing curricula.

Keywords
Research Design, Assessment, Qualitative Research, Quantitative Research, Research or Project Assessment

Educational Objectives

By the end of this activity, learners will be able to:

1. Describe the main elements of a research project.
2. Critically appraise the main aspects of a research project.
3. Develop action steps in SMART (Specific, Measurable, Achievable, Relevant, Time-bound) format.
4. Discuss the iterative nature of designing a research project.

Introduction

Health Professions Education Research (HPER) plays a pivotal role in improving the education of future health care providers and enhancing patient care and outcomes.[1] However, these benefits can be obtained only if HPER is developed and implemented rigorously.[2] Addressing HPER quality can be challenging since it possesses unique characteristics and principles that set it apart from traditional health care and basic science research. A key aspect of high-quality HPER is ensuring that research questions are clearly articulated and that project designs are well-aligned with these questions.[3] The most common reasons for journal editors to reject HPER manuscripts before external peer review are an unclear research question, inefficient project design, and suboptimal data collection process.[4]

In our literature search for evidence-based methodologies to train scholars in HPER planning, we identified workshops centered on some isolated aspects of HPER design, resources focused on performing a literature review and writing research questions,[5] theories and frameworks for HPER planning,[6] and incorporation of mixed methods in HPER.[7,8] Furthermore, these resources

Citation:
Harz D, Kesselheim J, Fischer K. The sailboat activity: an interactive, visually engaging approach to design and assess health profession education research projects. *MedEdPORTAL.* 2025;21:11520.
https://doi.org/10.15766/mep_2374-8265.11520

Copyright © 2025 Harz et al. This is an open-access publication distributed under the terms of the Creative Commons Attribution license.

mostly used case scenarios instead of engaging learners with their projects. With engagement, learners may feel more involved and responsible for creating each part of the research plan and may face the challenges of building a conceptual framework for their projects.[9]

For these reasons, we developed the interactive Sailboat activity, an innovative approach that goes beyond traditional workshops by actively engaging educators and scholars in refining their own HPER project plans, rather than working through predefined case scenarios, with specific focus on drafting and refining research questions and ensuring research design alignment. This activity fosters iterative critical appraisal, structured reflection, and collaborative problem-solving, addressing common pitfalls in HPER. We designed the Sailboat activity using principles from the agile retrospective framework, which is traditionally used in project management to promote continuous improvement through structured reflection.[10] The framework provides a structured, yet flexible approach in which participants assess their project's strengths, challenges, and actionable next steps. While traditionally used for retrospective analysis of completed work, we adapted this approach for HPER planning, allowing participants to critically examine and refine proposals at an early stage. By integrating reflective practice and project-based learning, the activity offers a novel way to enhance participants' ability to conceptualize, refine, and advance their research projects in a meaningful and practical manner.[9,11,12] Moreover, the visual and gamified elements encourage engagement and creativity, distinguishing this approach from conventional didactic methods. Through this process, participants gain experience in structuring high-quality HPER proposals, with particular emphasis on refining their research questions and their project's rationale and design. We implemented the Sailboat activity either in person or virtually at different institutions, and we herein present data from the local, virtual iteration of the workshop.

Methods

Background Context for Implementation

The workshop was offered for students in the Master of Medical Sciences in Medical Education (MMSc-Med Ed) program at Harvard Medical School in November 2021, 2022, and 2023, with 34 participants from 17 different health profession specialties (Table 1).

Table 1. Workshop Participants by Health Profession Discipline ($N = 34$)

Medical Disciplines	*n* (%)
Surgery	5 (15)
Emergency medicine	4 (12)
Pediatrics	4 (12)
Family medicine	4 (12)
Medical student	3 (9)
Medical education	2 (6)
General medicine	2 (6)
Internal medicine	1 (3)
Neonatology	1 (3)
Pathology	1 (3)
General dentistry	1 (3)
Maternal-fetal medicine	1 (3)
Psychiatry	1 (3)
Hematology	1 (3)
Geriatric and palliative medicine	1 (3)
Diagnostic medical sonographer	1 (3)
Clinical scientist	1 (3)

The MMSC-Med Ed is a 2-year program that aims to provide students with a solid foundation in the theories of HPE and engage participants in rigorous HPER. Participants enter their first semester of the program with varying degrees of previous knowledge and experiences with HPER. They learn about quantitative and qualitative methods that equip them with the foundations of developing an HPER project, including learning how to develop research questions and different research designs. The aim of the Sailboat activity is to enhance participants' skills in appraising HPER plans and to engage them in iterative planning. If this workshop were implemented in a group with no research experience, additional scaffolding—such as a more detailed introduction to HPER methodologies, guided facilitation during small-group discussions, or supplementary preliminary work—might be necessary to ensure productive discussions.

Sailboat Activity

We designed the Sailboat activity as a visually engaging exercise.[13] The Sailboat diagram has been used in fields outside of HPE as a brainstorming tool to collect feedback from teams about the state of a project, product, or organization. In our adaptation, we repurposed the Sailboat as a visual metaphor for an HPER project and designed the activity to help participants critically appraise and iteratively refine their research project ideas. This adaptation uniquely applies the Sailboat framework to HPER planning, integrating structured reflection, project-based learning, and gamification to foster engagement, dialogue, and iterative improvement and help educators progress toward the ultimate goal of designing a high-quality HPER proposal. The Sailboat represents the project; the goal is to move it to reach the island, which is the project's objective (Appendix A). The Sailboat could encounter challenges that hold it back or could face major problems that might entirely prevent it from arriving on the island. There may be positive elements that could drive the project forward. This activity focuses participants' attention on identifying impediments and beneficial aspects that can help them complete their project.[13]

Copyright © 2025 Harz et al. This is an open-access publication distributed under the terms of the Creative Commons Attribution license.

This project received institutional review board exemption from additional review by the Harvard Medical School Institutional Review Board, as it was classified as a course evaluation and quality improvement project. Therefore, written informed consent was not required. Participation in the survey was entirely voluntary, and respondents' identities were kept anonymous.

Preparatory Work

We uploaded all the instructions and materials to a learning management system for asynchronous preparation. However, these materials could also be shared via email or any communication method. We asked participants to complete a preworkshop reading[14] focused on writing a medical education research proposal. As a preworkshop assignment, participants had to craft a research plan using a Sailboat diagram (Appendix B).

To craft the research plan, participants were free to draw their Sailboat or were offered use of a Sailboat template that we created (Appendix A). We asked participants to include in the Sailboat the following components: characteristics of the research problem being addressed (sails), central research question (body of the boat), research design (flag), variables, outcome measures, population, sample, and source of data (windows), significance of the project for different audiences (island), research site (water), significant problems that may arise during the project (iceberg), obstacles (anchor), and positive elements as well as theoretical or conceptual frameworks that may help the development of the project (wind). To submit the assignment, we requested that participants upload it into an online collaborative platform and asked them to pose two questions about the project that they wanted to discuss during the synchronous workshop. As an alternative, participants could share their Sailboats via email. We asked participants to read each other's assignments before the workshop.

As part of their preparation for the 2022 and 2023 workshops, participants completed an anonymous, voluntary, 7-item survey on their perceived confidence about the different aspects of their research project. Response options, rated on a 5-point Likert scale, ranged from a minimum of 1 (*not at all confident*) to a maximum of 5 (*extremely confident*) on each of the 7 survey items (Appendix C). The preworkshop survey was hosted at Qualtrics. The authors developed the survey and aligned it with the workshop's educational objectives. The overall preparation for the workshop was no more than 1 hour.

During the Workshop

The workshop was 90 minutes long, held virtually via videoconferencing platform, and facilitated by two educators. The workshop facilitators need not be trained in HPER but would ideally have experience in HPER and the use of videoconferencing. The facilitator guide (Appendix D) provides guidelines for conducting the activity. The lead facilitator (Krisztina Fischer) explained the educational objectives, the time line of the session, and the details of the activity. The supporting facilitator (Dominique Harz) assisted with technology needs such as monitoring the chat and managing the breakout rooms. This workshop can also be held in person and thus be implemented with the primary facilitator only. For an in-person workshop, the facilitator would need whiteboards and markers.

Workshop Time Line

Introduction (15 minutes): The lead facilitator used PowerPoint slides to orient participants in the workshop's aims, methodology, and time line (Appendix E). In the meantime, the supporting facilitator created the breakout rooms, each composed of two participants. We designed a collaborative working area for every group that included each participant's Sailboat diagram and space to add aspects that could serve as obstacles (anchor), major problems (iceberg), and positive elements (wind) for the research plan (Appendix F). Additionally, space was left where participants could create an action plan related to their project.

Sailboat activity (40 minutes): Participants worked in pairs with their designated partner in the breakout rooms. They had 20 minutes each to present and discuss their project plan using their Sailboat diagram, divided into the following increments: 3 minutes to make a brief description of their project; 10 minutes to discuss its positive elements (wind), obstacles (anchors), and major problems (iceberg) and to address the presenter's questions; and 7 minutes to create an action plan for advancing the project. This action plan had to use objectives written in a SMART (Specific, Measurable, Achievable, Relevant, Time-bound) format.[15] We notified participants when they had 7 minutes remaining, to ensure that they completed their action plans.

Whole-group presentation (20 minutes): After the work in breakout rooms, all participants met in the main virtual room, and two participants voluntarily presented their project plan, along with a summary of strengths, weaknesses, and action steps. We encouraged the rest of the participants to share their opinions about the project's weaknesses and action steps. Our group size was between nine and 13 participants. However, the workshop can be implemented for much larger groups without changing the workshop's time line.

Group reflection (10 minutes): We asked participants to reflect on the Sailboat activity for 2 minutes and then complete the prompt

Copyright © 2025 Harz et al. This is an open-access publication distributed under the terms of the Creative Commons Attribution license.

"I used to think …" "Now I think …"[16] by writing their answers in the virtual chat. We then asked them to submit their responses by hitting send (designated the chat waterfall). After we received all responses on the chat waterfall, we read the messages out loud, and the group commented further, sharing insights on their experience.

Wrap up (5 minutes): We asked participants to complete a brief postworkshop survey (Appendix G).

Workshop Evaluation

In addition to the group reflection, participants completed an anonymous, voluntary postworkshop survey developed by the authors that included 11 items assessing participants' reactions (Kirkpatrick level 1)[17] to the activity and directly focused on the workshop's educational objectives. The response options, rated on a 5-point Likert scale, ranged from a minimum of 1 (*not at all useful*) to a maximum of 5 (*extremely useful*). The postworkshop survey included two open-ended questions about the workshop's strengths and potential areas for improvement (Appendix G).

Additionally, for 2022 and 2023, to assess the workshop's impact on participants' perceived confidence, the postworkshop survey included the same 7 items as the preworkshop survey asking participants to rate their perceived confidence level (Kirkpatrick level 2)[17] regarding their proposed project. The postworkshop survey was hosted at Qualtrics, and we shared its access link through the chat, which was also displayed as a QR code. Quantitative and qualitative descriptive analysis was used to provide a comprehensive description of the participants' experiences in the Sailboat activity.

After the workshop, the two facilitators scored participants' action plans using a scoring rubric developed by the authors (Appendix H). Quality of the action plan's objective was assessed on a 3-point scale based on the inclusion of the SMART framework elements (1 = *vague*, 2 = *partial inclusion*, 3 = *full inclusion*), and alignment of the action plan with the project's weaknesses was assessed on a 2-point scale (1 = *not aligned*, 2 = *aligned*; Kirkpatrick level 2).[17]

Results

Quantitative Results

To determine differences in participants' perceived confidence about their project before and after the workshop, we assessed the 7 items included in the pre- and postworkshop surveys: (1) barriers, (2) goals, (3) strengths, (4) weaknesses, (5) main elements, (6) alignment of the main elements, and (7) next steps of their proposed research project. The preworkshop survey response rate was 84% (21/25). Confidence scores for the 7 preworkshop survey items ranged from $M = 3.2$ ($SD = 0.6$) to $M = 3.6$ ($SD = 0.6$). The postworkshop survey response rate was 91% (31/34). Confidence scores for the 7 postworkshop survey items ranged from $M = 3.7$ ($SD = 1.0$) to $M = 4.1$ ($SD = 1.0$). Based on a two-sample *t* test at a .05 significance level, we found a statistically significant increase in participants' perceived confidence about their project from pre- to postworkshop. Participants' confidence ratings in response to all 7 survey items are summarized in Table 2.

Participants' reactions to the Sailboat activity were evaluated using an 11-item postworkshop survey. Participants' ratings for each of these 11 survey items are summarized in the Figure.

Table 2. Comparison Between Participants' Perceived Confidence Pre- and Postworkshop

Survey Item[a]	Preworkshop Rating *M (SD)*[b]	Postworkshop Rating *M (SD)*[c]	*p*
As you look at your Sailboat now, how confident do you feel about:			
The barriers of your research project?	3.2 (0.8)	3.9 (0.9)	.008[d]
The goal(s) of your research project?	3.6 (0.6)	3.9 (1.0)	.10
The strengths of your research project?	3.4 (0.7)	3.7 (1.0)	.10
The weaknesses of your research project?	3.2 (0.6)	4.1 (1.0)	.001[d]
The project's main elements (problem, research question, objectives, type of study, variables, population and sample, and outcome measures)?	3.3 (0.7)	3.9 (1.0)	.04[d]
The alignment of the main elements (problem, research question, objectives, type of study, variables, population and sample, and outcome measures) of your research project?	3.3 (0.7)	3.8 (1.0)	.03[d]
The next steps in your project development?	3.2 (0.8)	4.1 (0.8)	.001[d]

[a]Rated on a 5-point Likert scale (1 = *not at all confident*, 5 = *extremely confident*).
[b]The preworkshop survey response rate was 84% (21/25).
[c]The postworkshop survey response rate was 91% (31/34).
[d]Significant at $p < .05$.

Copyright © 2025 Harz et al. This is an open-access publication distributed under the terms of the Creative Commons Attribution license.

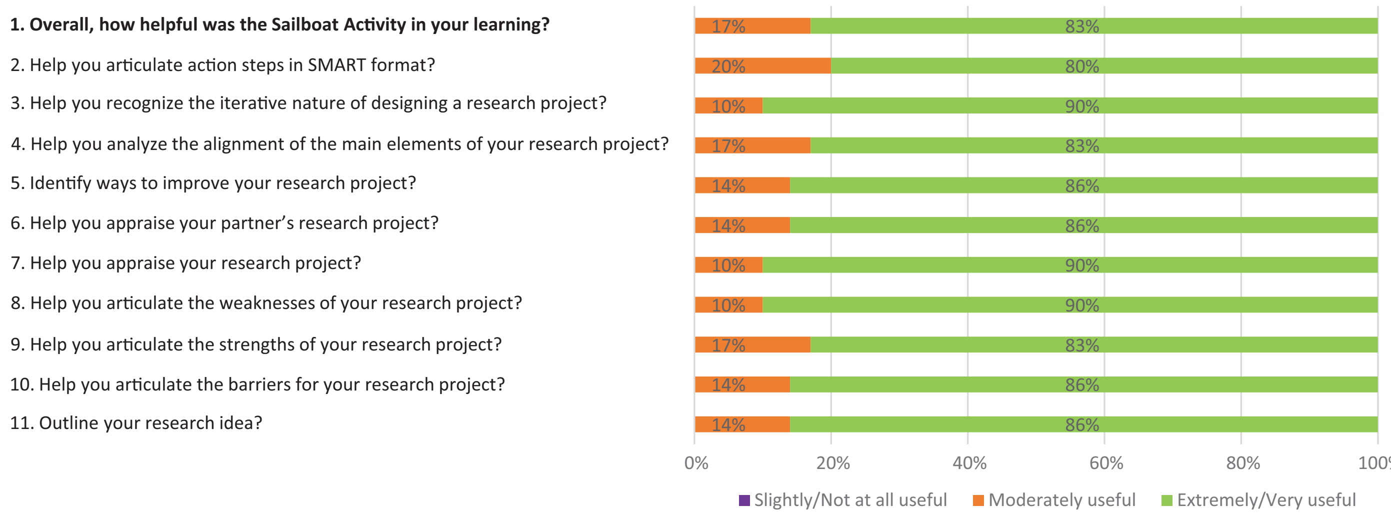

Figure. Distribution of participants' ratings on the postworkshop survey ($N = 31$). Items 2 to 11 used the same question stem: "How useful was the Sailboat activity to:". Items were rated on a 5-point Likert scale (1= *not at all useful*, 5 = *very useful*). Abbreviation: SMART, Specific, Measurable, Achievable, Relevant, Time-bound.

The quality of participants' action plans based on inclusion of the SMART criteria and alignment of the participants' SMART action plans with their projects' weaknesses were evaluated by two facilitators after the workshop (Appendix H). The two facilitators' ratings were as follows: for quality of action plan, $M = 2.4$ ($SD = 0.6$); for alignment, $M = 1.8$ ($SD = 0.4$). The interrater reliability between the two facilitators, assessed using Cohen's kappa, was found to be 0.81 for quality of the action steps, and 0.85 for alignment with the project's weaknesses.

Qualitative Responses

During the group reflection at the end of the workshop, when participants were asked to respond to the prompt "Now I think …," 82% of participants (28/34) completed it. They reflected on how the activity helped them better understand the nuances of HPER planning and the value of critically appraising the project plan with fellow educators and scholars (Table 3). Additionally, some participants recognized how discussing a project can benefit them by improving their feasibility and success (Table 3).

In participants' responses to the open-ended question 1 ("What were the strengths of the Sailboat activity?") included in the postworkshop survey, participants' overall opinion about the activity was related to the benefit of using a visual format to present the projects. First, displaying their projects as a Sailboat made them include a project's different components in one image. Participants indicated that use of the Sailboat to visualize their projects not only helped them organize their ideas about their projects but also made it easier to understand their classmates' research plans, thus facilitating feedback and collaboration among their peers. They also appreciated the collaborative nature of the activity, where they could offer feedback to their colleagues and gather some new ideas to advance their projects (Table 3).

In participants' responses to the open-ended question 2 ("What could have gone better in the Sailboat Activity?") in the postworkshop survey, some participants suggested that the time spent discussing the projects with the whole class should be increased or that more participants should be added to the small-group conversation. In addition, there were a few comments about technology challenges. Finally, one participant mentioned that they thought the activity worked well and that they would not change anything (Table 3).

Discussion

The Sailboat activity proved to be an effective and engaging tool for fostering critical appraisal and iterative refinement of HPER project plans. Beyond its feasibility in a virtual environment, the activity provided a structured, yet flexible approach that encouraged participants to reflect on their research questions, identify gaps in their project designs, and take actionable steps toward improving their projects. By actively engaging in project-based learning, participants not only strengthened their individual research proposals but also gained insight into common challenges in HPER, such as aligning project design with research objectives and articulating clear next steps for project development.

Copyright © 2025 Harz et al. This is an open-access publication distributed under the terms of the Creative Commons Attribution license.

Table 3. Participant Responses to Survey Prompts and Open-Ended Questions for the Sailboat Activity

Prompt/Question	Theme	Representative Quotes From Sailboat Activity Workshop Participants
"I used to think ..." "Now I think ..."	The iterative and multifaceted nature of HPER planning	"I used to think mostly about the research question, variables, and outcomes. Now I think more broadly and comprehensively about all the different elements of a robust study"—P2, 2021<br>"I used to think creating a study design was relatively straightforward. Now I think I have a lot more reflection to do on how to best design/create my research project using good framework/theory"–P6, 2021<br>"I used to think designing medical education research was straightforward and clear cut, and now I think (realize) that there are many intricacies and complexities that go into design that affect everyone designing ME research questions, not just me"–P12, 2022
	The value of discussion and critical appraisal of an HPER project	"I used to think my project was too narrow and didn't have a good outcome measure. Now I think I need to select a measure and stick with it."–P7, 2021<br>"I used to think that it is very hard to do research, and now I think that with proper support [it] is feasible."–P14, 2022<br>"I used to think research question development was more of an individual's responsibility to create. Now I think that a community of scholars can help refine and elevate your question."–P16, 2022<br>"I used to think that creating research plans was stressful and overwhelming. By breaking it down into the elements of a 'sailboat,' I now see it as much more manageable."–P2, 2023
What were the strengths of the Sailboat activity?		"Putting all the concepts and updates of my project in one place, easy to find what I'm looking for"–P8, 2021<br>"Taking the time to make the sailboat individually was really useful. I'm going to use a sailboat now when I design future research projects."–P14, 2022<br>"Easy to buy-in to partner's sailboat and give feedback"–P1, 2021<br>"Good collaboration between classmates to help everyone's project"–P5, 2021<br>"Pair discussion on next steps"–P19, 2022<br>"It really made you think critically about the project, identifying barriers."–P1, 2023<br>"It is a new, creative way of thinking; breaking it down into smaller components was helpful; I actually took my sailboat and made it into a word document that I shared [with] my possible mentor."–P2, 2023<br>"Receive helpful feedback from your peers."–P8, 2023<br>"It was fun to get creative and artistic with developing my sailboat!"–P9, 2023
What could have gone better in the Sailboat activity?		"Opportunity for everyone to get feedback on their sailboats from the whole class"–P2, 2021<br>"I would have liked to review the sailboats in groups of three instead of pairs, just to get additional feedback and create more conversation about the projects"–P14, 2022<br>"I know timing is hard, but it would have been nice to meet with 2 partners to get different perspectives."–P1, 2023<br>"I think rotating us through talking with 2 people to get more feedback would be even better. Spending less time with each person, but getting more eyes on it."–P8, 2023<br>"Computer operation is somewhat complicated"–P11, 2022<br>"Nothing! Love the feedback I got and gave"–P1, 2021<br>"All went very well"–P19, 2022

Abbreviation: HPER, health professions education research; P, participant.

A key takeaway from the activity was its role in building confidence and enhancing participants' ability to critically evaluate their own and others' work in a collaborative setting. Participants reported that the visual and structured nature of the activity helped them think more comprehensively about their projects, reinforcing the iterative nature of research planning and the importance of peer feedback in HPER development. These findings suggest that the Sailboat activity can play a valuable role in improving research planning skills, which are critical for producing rigorous and impactful HPER.

Using the feedback we received from participants in the first iteration of the workshop, we made several changes in the implementation of the Sailboat activity. For example, participants consistently stated that they appreciated how the activity improved their project designs by helping them articulate the project's strengths and weaknesses and move the project further by creating effective SMART action steps. Despite the fact that participants provided positive feedback and identified high-quality SMART action steps during the first iteration of the workshop, we were unsure if participants gained confidence in their HPER project planning skills. In the second iteration of the Sailboat activity, we added 7 survey items pre- and postworkshop that assessed participants' confidence levels in their project plan development. Participants' confidence level in identifying their projects' weaknesses and next steps was significantly higher after the Sailboat activity.

The Sailboat activity was a valuable tool for meeting the workshop's educational objectives, illustrated by the participants' comments and their in-class work. Participants described how the activity helped them appraise the research idea outline and alignment of the project's components, both visually and cognitively. They also explained how, during the workshop, they were able to think more comprehensively about their projects, to recognize the iterative nature of designing a project, and to appreciate the unique nuances of HPER research.

Copyright © 2025 Harz et al. This is an open-access publication distributed under the terms of the Creative Commons Attribution license.

Participants frequently requested more time to discuss each participant's project plan within the large group in their feedback. Therefore, we will add 30 minutes for future iterations to accommodate everyone who wants to present.

Some of the participants mentioned a desire for an additional participant to be included in the small-group project consultation in order to collect more feedback on their HPER project plan. Although we were unable to add an additional participant to the breakout room discussion, given the available time for our activity, other faculty members conducting the Sailboat activity may consider increasing the small-group size and adding 30 minutes to the activity, if time allows.

We encountered several limitations. There were 17 health profession specialties represented in our group of participants. While the diverse expertise in the large group provided opportunities for rich discussions and building on each other's prior experience, not all of our participants could evaluate the nuances of each project. In addition, only two volunteers could present in the large group as part of the 2-hour workshop. Furthermore, most of our evaluation focused on Kirkpatrick level 1 and level 2 outcomes.[17] Future work will need to assess higher-level outcomes; for example, one could follow up and see if the projects were implemented and completed (level 3 outcome).

Overall, the findings related to the Sailboat activity offer promising insights into its potential as an effective tool for enhancing HPER project planning skills, increasing learner confidence, and fostering a collaborative and engaging learning environment in HPER. The activity successfully addressed the identified gap in HPER training by providing a structured, interactive, and participant-driven approach to refining research questions and crafting and aligning research designs. Unlike traditional workshops that focus on isolated aspects or research planning, the Sailboat activity engaged participants directly with their own projects, fostering both iterative planning and confidence in HPER development. These aspects are well-aligned with the expectations for rigorous HPER, which emphasize the importance of critically appraising HPER plans, assessing the quality of these plans through multiple dimensions, and engaging in critical discussion with colleagues.[18,19] The Sailboat activity can be adapted to other educational environments, and incorporated into existing curricula in a range of training programs with multiple disciplines at various training levels.

Appendices

A. Sailboat Template.pptx
B. Preworkshop Assignment Instructions.docx
C. Preworkshop Survey.docx
D. Facilitator Guide.docx
E. Sailboat Activity Session Slides.pptx
F. Collaborative Working Area.pptx
G. Postworkshop Survey.docx
H. Action Plan Scoring Rubric.docx

All appendices are peer reviewed as integral parts of the Original Publication.

Dominique Harz, DMD, MDS, MMSc: Instructor of Orthodontics, Pontificia Universidad Católica de Chile

Jennifer Kesselheim, MD, MEd: Associate Professor of Pediatrics and Designated Institutional Official, Boston Children's Hospital and Harvard Medical School

Krisztina Fischer, MD, PhD, MMSc: Associate Professor of Radiology, Brigham and Women's Hospital; Faculty Director, Master of Medical Sciences in Medical Education, Harvard Medical School; ORCID: https://orcid.org/0000-0003-4471-2918

Disclosures
We are very grateful to the classes of 2023, 2024, and 2025 Harvard Medical School Master of Medical Sciences in Medical Education students for participating in this workshop.

Funding/Support
None to report.

Ethical Approval
The Harvard Medical School Institutional Review Board reviewed this project.

References

1. Abramson EL, Paul CR, Petershack J, et al. Conducting quantitative medical education research: from design to dissemination. *Acad Pediatr*. 2018;18(2):129-139. https://doi.org/10.1016/j.acap.2017.10.008
2. Gruppen LD. Improving medical education research. *Teach Learn Med*. 2007;19(4):331-335. https://doi.org/10.1080/10401330701542370
3. Bordage G. Reasons reviewers reject and accept manuscripts: the strengths and weaknesses in medical education reports.

Copyright © 2025 Harz et al. This is an open-access publication distributed under the terms of the Creative Commons Attribution license.

Acad Med. 2001;76(9):889-896. https://doi.org/10.1097/00001888-200109000-00010

4. Meyer HS, Durning SJ, Sklar DP, Maggio LA. Making the first cut: an analysis of academic medicine editors' reasons for not sending manuscripts out for external peer review. *Acad Med*. 2018;93(3):464-470. https://doi.org/10.1097/ACM.0000000000001860
5. Lebeau R. Guiding educational research projects: activity-based workshops on writing a literature review and developing research questions. *MedEdPORTAL*. 2015;11:10143. https://doi.org/10.15766/mep_2374-8265.10143
6. Rougas S, Berry A, Bierer SB, et al. Applying conceptual and theoretical frameworks to health professions education research: an introductory workshop. *MedEdPORTAL*. 2022;18:11286. https://doi.org/10.15766/mep_2374-8265.11286
7. Schifferdecker K. When quantitative or qualitative data are not enough: application of mixed methods research in medical education. *MedEdPORTAL*. 2008;4:1146. https://doi.org/10.15766/mep_2374-8265.1146
8. Blanchard R, Scott J. Connecting mixed methods as an education research strategy. *MedEdPORTAL*. 2014;10:9670. https://doi.org/10.15766/mep_2374-8265.9670
9. Guo P, Saab N, Post LS, Admiraal W. A review of project-based learning in higher education: student outcomes and measures. *Int J Educ Res*. 2020;102:101586. https://doi.org/10.1016/j.ijer.2020.101586
10. Przybyłek A, Albecka M, Springer O, Kowalski W. Game-based Sprint retrospectives: multiple action research. *Empir Softw Eng*. 2022;27:1. https://doi.org/10.1007/s10664-021-10043-z
11. Schon DA. *The Reflective Practitioner: How Professionals Think in Action*. Basic Books; 1984.
12. Loyens SMM, Wijnia L, Rikers RMJP. Student-centered instruction: inquiry-, problem-, project-, and case-based learning. In: Tierney RJ, Rizvi F, Ercikan K, eds. *International Encyclopedia of Education*. 4th ed. Elsevier; 2023:701-711. https://doi.org/10.1016/B978-0-12-818630-5.14080-1
13. Gonçalves L, Linders B. *Getting Value Out of Agile Retrospectives. A Toolbox of Retrospective Exercises*. lulu.com; 2014.
14. Fischer K, Kesselheim JC. 23 questions to guide the writing of a quantitative medical education research proposal. *Acad Med*. 2022;97(1):164. https://doi.org/10.1097/ACM.0000000000003660
15. Doran GT. There's a S.M.A.R.T. way to write management's goals and objectives. *Manag Rev*. 1981;70(11):35-36.
16. Project zero: visible thinking. Harvard Graduate School of Education. Accessed March 21, 2025. https://pz.harvard.edu/projects/visible-thinking
17. Kirkpatrick JD, Kirkpatrick WK. *Kirkpatrick's Four Levels of Training Evaluation*. ATD Press; 2016.
18. O'Brien BC, Rees CE, Palermo C. Chapter 4. Quality in health professions education research. In: Rees CE, Monrouxe LV, O'Brien BC, Gordon LJ, Palermo C, eds. *Foundations of Health Professions Education Research: Principles, Perspectives and Practices*. John Wiley & Sons, Ltd; 2023:58-83.
19. O'Brien BC, West CP, Coverdale JH, Durning SJ, Roberts LW. On the use and value of reporting guidelines in health professions education research. *Acad Med*. 2020;95(11):1619-1622. https://doi.org/10.1097/ACM.0000000000003666

Received: October 9, 2024
Accepted: March 17, 2025
Published: May 2, 2025

Copyright © 2025 Harz et al. This is an open-access publication distributed under the terms of the Creative Commons Attribution license.
