# Supplementary figures and images for: The Sailboat Activity: An Interactive, Visually Engaging Approach to Design and Assess Health Profession Education Research Projects
Source: MedEdPORTAL. 2025 May 2;21:11520. doi: 10.15766/mep_2374-8265.11520 (PMC12046060; doi:10.15766/mep_2374-8265.11520)

## Slide 1
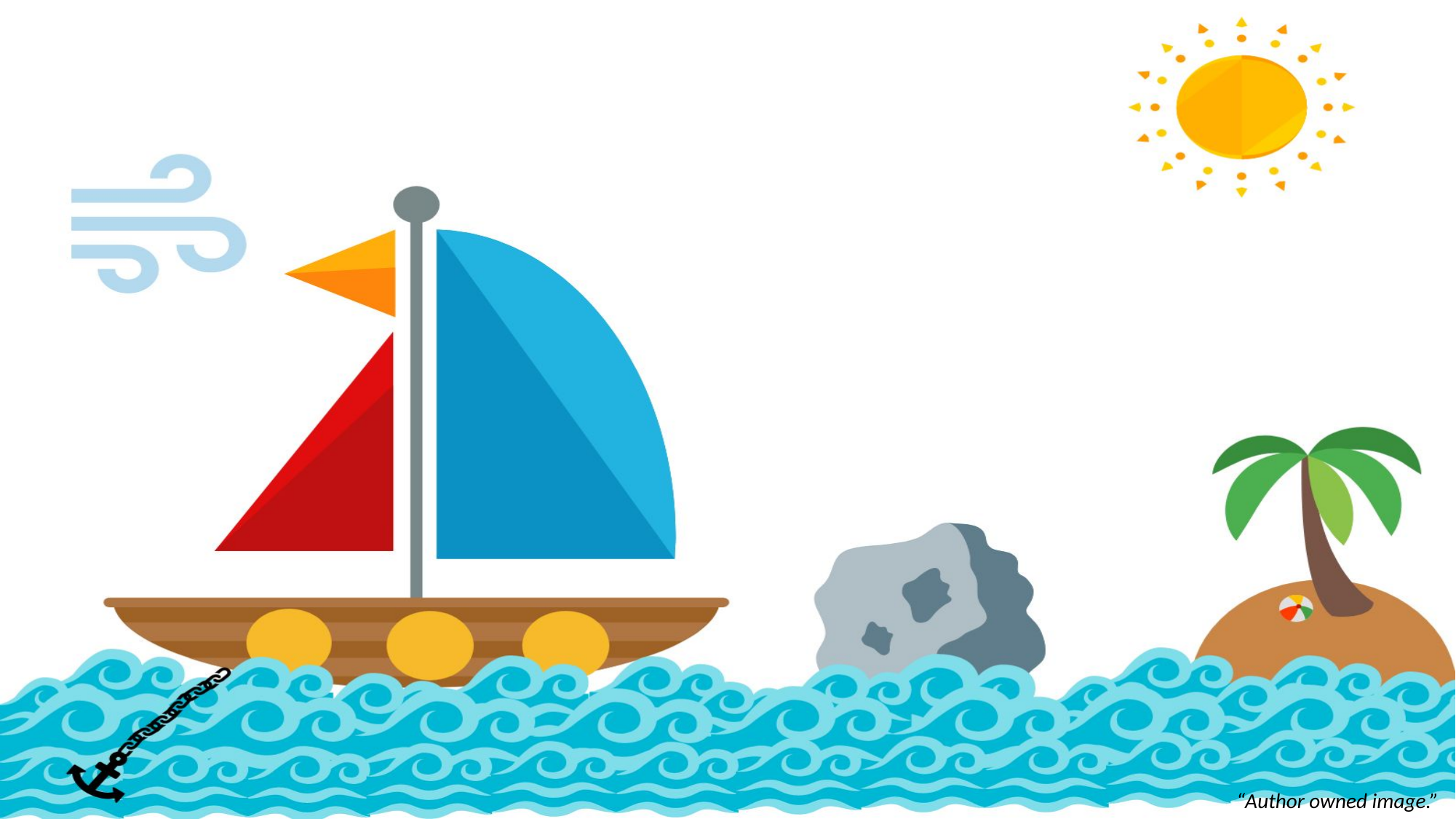

“Author owned image.”

## Slide 2
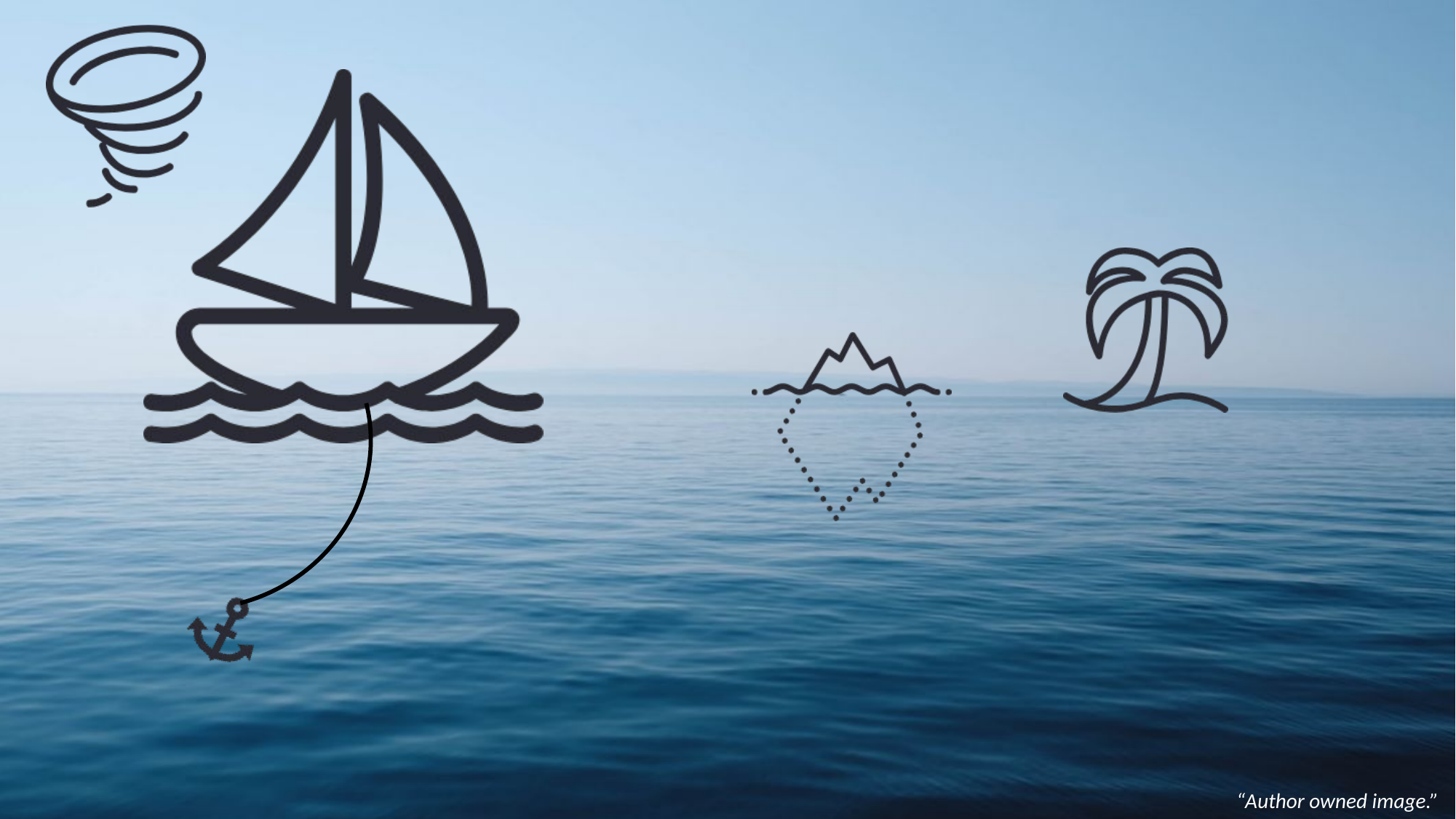

“Author owned image.”

Supplement: Supplementary file 1 — Sailboat Template.pptxPreworkshop Assignment Instructions.docxPreworkshop Survey.docxFacilitator Guide.docxSailboat Activity Session Slides.pptxCollaborative Working Area.pptxPostworkshop Survey.docxAction Plan Scoring Rubric.docx [file mep_2374-8265.11520-s001.zip › A. Sailboat Template.pptx]
